# A Single Interfacial Point Mutation Rescues Solution Structure Determination of the Complex of HMG-D with a DNA Bulge

**DOI:** 10.1002/cbic.202400395

**Published:** 2024-11-07

**Authors:** Guy R. Hill, Ji-Chun Yang, Laura E. Easton, Rachel Cerdan, Stephen H. McLaughlin, Katherine Stott, Andrew A. Travers, David Neuhaus

**Affiliations:** a MRC Laboratory of Molecular Biology, Francis Crick Avenue, Cambridge CB2 0QH, UK; b LPHI, Univ Montpellier, CNRS, Inserm, Place Eugène Bataillon, 34095 Montpellier, France; c Department of Biochemistry, University of Cambridge, 80 Tennis Court Road, Cambridge CB2 1GA, UK

**Keywords:** DNA recognition, HMG-box protein, NMR spectroscopy, Protein-DNA complex, MR linewidth

## Abstract

Broadening of signals from atoms at interfaces can often be a limiting factor in applying solution NMR to the structure determination of complexes. Common contributors to such problems include exchange between free and bound states and the increased molecular weight of complexes relative to the free components, but another cause that can be more difficult to deal with occurs when conformational dynamics within the interface takes place at an intermediate rate on the chemical shift timescale. In this work we show how a carefully chosen mutation in the protein HMG-D rescued such a situation, making possible high-resolution structure determination of its complex with a dA_2_ bulge DNA ligand designed to mimic a natural DNA bend, and thereby revealing a new spatial organization of the complex.

## Introduction

Solution NMR remains one of the most powerful methods available for determining structures of complexes of proteins, particularly for systems where binding is relatively weak and which are too small for electron microscopy and/or too flexible for X-ray crystallography to work well. Different NMR-based strategies are available that can yield different levels of structural information depending on the input data, but by far the richest and most detailed source of high-resolution information, when available, comes from the measurement of assigned, intermolecular NOE interactions that provide specific atom-to-atom close contact distance restraints across interfaces. However, the NMR properties of complexes in solution cannot be taken for granted, and in many cases such studies are made difficult or frustrated completely by line-broadening effects that render intermolecular NOE interactions undetectable. Such line-broadening can have several distinct origins. Exchange between free and bound states under conditions when both are present and the exchange rate falls in the “intermediate rate regime” (k_exch_ ≈ Δδ) may selectively broaden signals from interfacial atoms whose chemical shifts are altered in the complex; in addition, rapid relaxation will broaden all signals in high molecular weight or aggregated systems. However, another likely source is intermediate rate conformational dynamics at the interface, and unlike free-bound exchange this cannot be ameliorated by optimising the relative concentrations of the components. Here we present an example of such a system, a complex of the protein HMG-D with a bulge DNA ligand that mimics a natural DNA bend, where these properties originally caused very severe difficulties during attempted structure determination leading to a probably flawed model,^[1]^ and we show how a rational point mutation corresponding to deletion of a single atom (Tyr mutated to Phe) subsequently transformed the NMR properties of the interface, allowing a high-resolution solution structure to be determined.

High-mobility group box (HMGB) proteins are essential and ubiquitous proteins that often have multiple functions, playing key roles both within the nucleus, where they are involved in DNA organisation and compaction as well as regulation of transcription, replication, recombination, chromatin remodelling and DNA repair,^[2]^ and extracellularly, where they can act to modulate immune signalling and inflammatory responses.^[3]^ The distinguishing structural feature of HMGB proteins is the presence of one or more HMG box domains, an L-shaped domain of three α-helices that, together with unfolded tails and/or linkers, is responsible for the DNA binding properties of these proteins. Two classes of HMGB proteins can be distinguished, according to whether DNA binding is sequence-specific, as for example with transcription factors such as LEF1,^[4]^ SRY^[5]^ and SOX5,^[6]^ or non-sequence-specific, as for example with the mammalian chromosomal proteins HMGB1 and HMGB2,^[7]^ their counterparts in *Drosophila melanogaster*, HMG-D^[8]^ and HMG-Z,^[9]^ and the *Saccharomyces cerevisiae* protein NHP6A.^[10]^ As part of early studies to investigate the function of HMG-D and in particular its potential roles in recognising, binding to and/or facilitating the formation of specific architectural features in DNA,^[11]^ in 1994 the Neuhaus group published a solution structure of the box domain (residues 2–74) of HMG-D,^[12]^ followed in 2001 by a structural model of the complex between full-length HMG-D and a 14:12 dA_2_ bulge DNA ligand designed to mimic a natural DNA bend (PDB 1E7J).^[1]^ However, investigation of this complex was severely hindered by extensive line-broadening of NMR signals from the interfacial region, with the consequence that only a very small number of intermolecular NOE contacts could be identified. Differences in solution conditions, protein:DNA ratio and temperature did not alter this situation, and we hypothesised that the recalcitrant nature of the problem might be caused by intermediate-rate internal dynamics within the structure of the interface itself.

More recently we returned to this problem, to test whether making a small change to the system might modify the dynamics of the interface in such a way as to reduce or eliminate the line-broadening. We had previously speculated that Tyr12 might be involved in these effects, given its central location within the protein-DNA interface.^[1]^ Positional changes of aromatic rings during dynamic processes at the interface might be associated with large, ring-current-induced chemical shift changes for neighbouring spins, and involvement of the Tyr sidechain OH group in one or more hydrogen bonds might possibly retard such movements sufficiently to bring them into the intermediate exchange rate regime for chemical shifts. Note that ring-flip motions of axially symmetric aromatic rings are not relevant here, since they can only modulate chemical shifts of spins on the ring itself, not those in its surroundings. To cause broadening for *neighbouring* spins, movement of an aromatic ring must connect states where the starting and final positions of the ring differ relative to its environment, which is not the case for the flip of a symmetrical ring in Phe or Tyr. As elaborated further in the Results and Discussion section, we speculate that the actual exchange process responsible might involve minor populations in which protein is translocated on the DNA, a process that has been demonstrated experimentally for DNA binding of another non-sequence-specific HMG protein, HMGB-1 A.^[13]^ Such a process might occur in both the WT and Y12F HMG-D complexes, but only in the case of the WT complex would the exchange potentially be slowed by intermolecular hydrogen-bonding interactions; in the Y12F mutant faster exchange could move the system into the fast-exchange regime, thereby eliminating the broadening.

**Figure 1:**
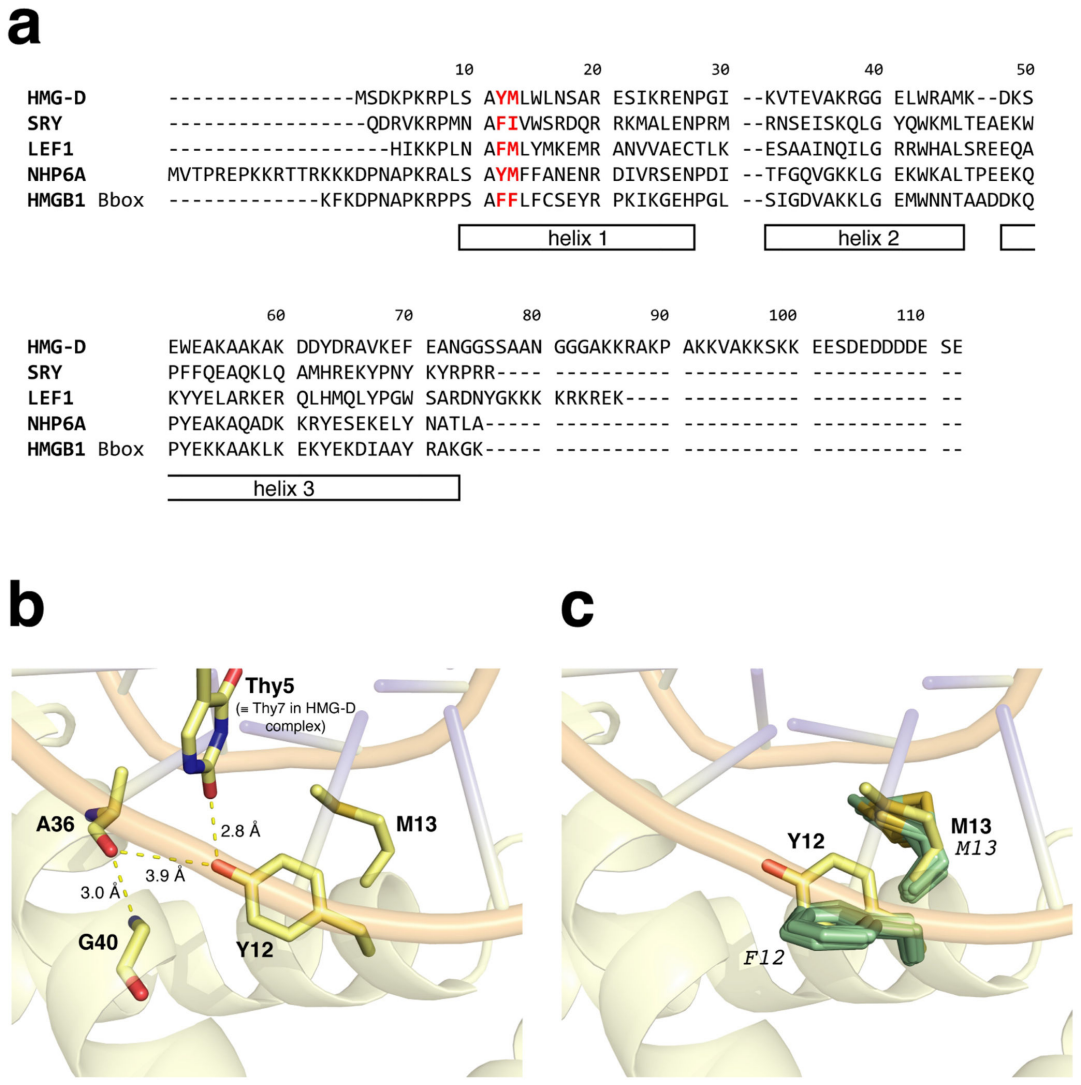
Variations in residues 12 and 13 of HMG box domains. a) Sequence alignment of HMG box domains for which an NMR structure of a complex with DNA has been deposited. Residues at positions 12 and 13 are highlighted in red. The numbering and helix locations shown are for HMG-D. PDB codes for the corresponding structures are as follows: HMG-D: 1E7J (original paper^[1]^), 8R1X (this work); SRY: 1HRY, 1HRZ,^[15a]^ 1J46^[15b]^; LEF1: 2LEF^[16]^; NHP6A: 1J5N^[10,14]^; HMGB1 Bbox, 2GZK.^[17]^ b) Hydrogen bonding network involving the sidechain OH group of Tyr12 as seen in the crystal structure (1QRV) of HMG-D WT in complex with a linear DNA. In the second copy of the HMG-D box domain present in the 1QRV crystal structure, for which interaction with the DNA is minimal and does not involve Tyr12, the H-bond between the Tyr12 sidechain OH group oxygen and Ala36 carbonyl shortens slightly to 3.7 Å. c) Superposition showing the sidechain conformations of Tyr12 in 1QRV and Phe12 in the NMR ensemble shown in Figure 3 of the complex of HMG-D Y12F bound to the dA_2_ bulge DNA ligand. Structures were superposed using the backbone atoms (N, C^α^, C’) of residues 5–70 of the protein.

Consistent with the suggestion that Tyr12 might be associated with such line-broadening, comparison with other DNA complexes of HMG box domain proteins for which NMR-based structures have been deposited revealed that in all but one of the other cases (NHP6A^[10,14]^) that we are aware of (complexes of SRY,^[15]^ LEF1^[16]^ and the B box of HMGB1 in a tandem construct with SRY^[17]^), the residue homologous to Tyr12 of HMG-D is a phenylalanine (Figure 1a), and in each of these cases a substantial number (>50) of intermolecular NOE contacts across the protein-DNA interface were detected and assigned (see Supp. Table S1). Additional support comes from the observations that exchange line-broadening of interfacial protein NMR signals has also been reported for complexes of HMG-D (2–100) with a disulphide cross-linked DNA,^[18]^ and that in the crystal structure of HMG-D (2–74) bound to a different (linear) DNA,^[19]^ the sidechain OH of Tyr12 forms an intermolecular H-bond, in that instance to O2 of a thymidine base in the DNA (Figure 1b).

## Results and Discussion

**Figure 2:**
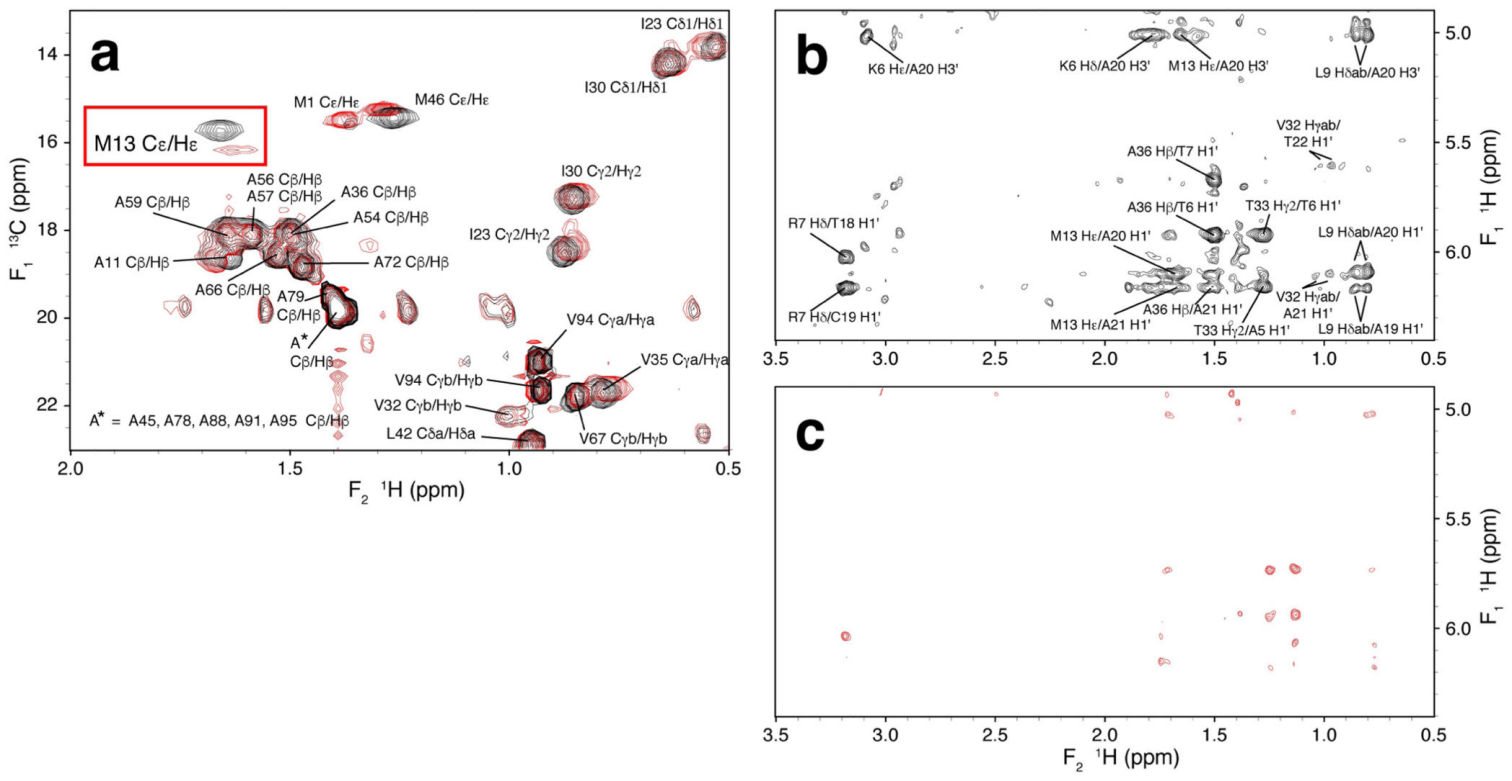
Effect of the Y12F mutation on NMR spectra of the HMG-D–dA_2_ DNA bulge complex. In all panels, signals of the HMG-D Y12F mutant complex are shown in black, signals of the wild-type HMG-D complex in red; all spectra were recorded at 800 MHz and 27 °C from samples of complexes containing [^13^C,^15^N] labelled protein and natural abundance DNA (chemical shift assignments have been deposited at the BMRB database under accession codes 34874 for the DNA complex of HMG-D Y12F and 52208 for the backbone signals of free HMG-D Y12F). a) Part of the methyl region of [^13^C, ^1^H] HSQC spectra of the complexes of HMG-D Y12F and HMG-D WT with the 12:14 dA_2_ DNA bulge ligand, showing selected assignments. The height of the Met13 C^ε1^/H^ε1^ signal for the Y12F mutant is approximately 4 times greater than that for WT. Contour levels in the two plots are normalised using signals well removed from the protein:DNA interface; see Supporting Information for further details. b) Part of the filtered NOESY spectrum (τ_m_ = 200 ms) of the complex of HMG-D Y12F with the 12:14 dA_2_ DNA bulge ligand. Filter elements in the pulse sequence were set to retain signals from ^12^C-bound (i. e. DNA) protons in F_1_ and ^13^C-bound (i. e. protein) protons in F_2_, thereby selecting for intermolecular NOE cross peaks; selected assignments are shown. c) Part of the corresponding spectrum of the complex of HMG-D WT with the 12:14 dA_2_ DNA bulge ligand. Contour levels and numbers of acquired transients in b) and c) were normalised to reflect the concentration ratio between the two samples in these two spectra, all other acquisition parameters being identical between the two; see Supporting Information and Supp. Figure S1 for further details

**Figure 3:**
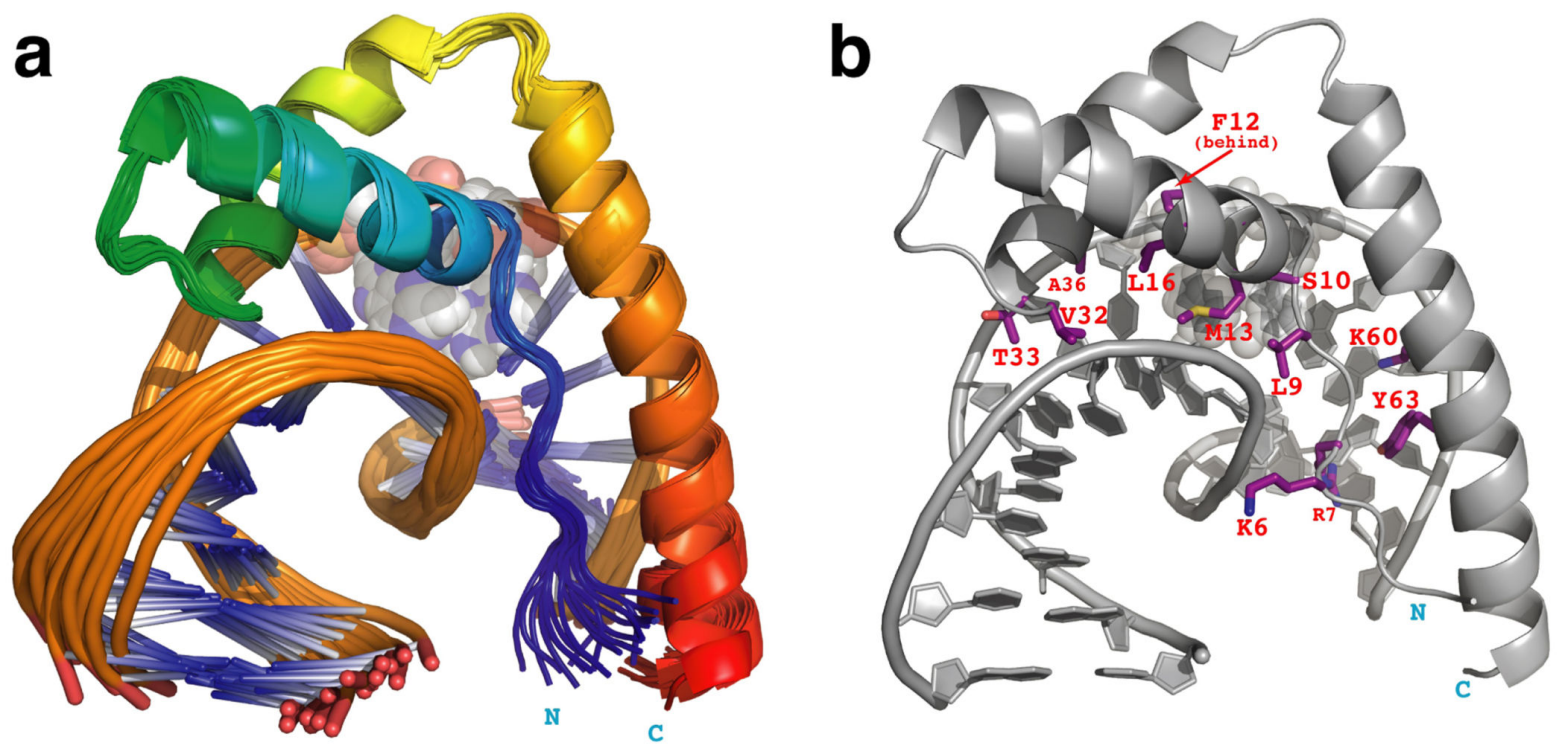
Structure of HMG-D Y12F bound to a 12:14 dA_2_ DNA bulge ligand. Atoms of the dA_2_ bulge nucleotides (A8 and A9) are shown as translucent spheres throughout (co-ordinates and structural restraints for this ensemble have been deposited with the PDB database under accession code 8R1X). a) Cartoon representation of the deposited ensemble of the 20 NMR structures of the HMG-D Y12F – DNA complex (superposed using the backbone atoms of protein residues 5–70 and DNA nucleotides 4–13 and 16–23; see Table 1 for structural statistics). The protein backbone is shown in “chainbow” colouring (blue → red, N-terminal → C-terminal). b) Cartoon representation of the lowest energy structure of the HMG-D Y12F – DNA complex, showing in purple the sidechains of those interfacial protein residues for which intermolecular NOE interactions were detected.

Based on this reasoning, we therefore prepared the Y12F point-mutant of full-length HMG-D and followed essentially the same strategy as in our earlier publication^[1]^ to determine the structure of its complex with the same dA_2_ 12:14 bulge DNA as had been used for the wild-type (WT) complex; in addition, we acquired fresh, corresponding data for the WT complex under near-identical conditions (the only difference was a slightly higher measured concentration for the Y12F complex) and employing identical NMR hardware, so as to allow for a carefully controlled comparison between the two cases, isolating the Y12F mutation as the sole reason for differences between the spectra. At an overall level, spectra of the Y12F complex appear qualitatively similar to those from the WT complex, aside from detailed differences in chemical shifts as would be expected (see Supp. Figures S1 and S2); some of these differences are likely caused by a change in the orientation of the phenyl ring of residue 12 between the WT and Y12F complexes (Figure 1c). However, as may be seen very clearly in Figure 2, signals from the interfacial region are significantly weaker in the spectra from the WT complex than in those from the Y12F complex (contour levels in these plots were set to facilitate comparison by correcting for the difference in sample concentrations; see supplementary information methods section). In the [^13^C,^1^H] HSQC spectrum (Figure 2a), the methyl signal of Met13, which like Tyr12 is centrally located within the interface, shows a much-reduced intensity in the WT case relative to the Y12F spectrum, and, crucially, the intensity of intermolecular NOE cross peaks in filtered NOESY spectra of the Y12F complex (Figure 2b) are drastically improved relative to the much weaker signals seen in corresponding spectra from the WT complex (Figure 2c). As a direct consequence of this improvement, we were able to identify a total of 65 intermolecular NOE restraints (see Supp. Table S2). These were used as part of the input to the structure calculations that resulted in the well-defined ensemble shown in Figure 3, with structural statistics shown in Table 1 and Supp. Figure S3.

**Figure 4:**
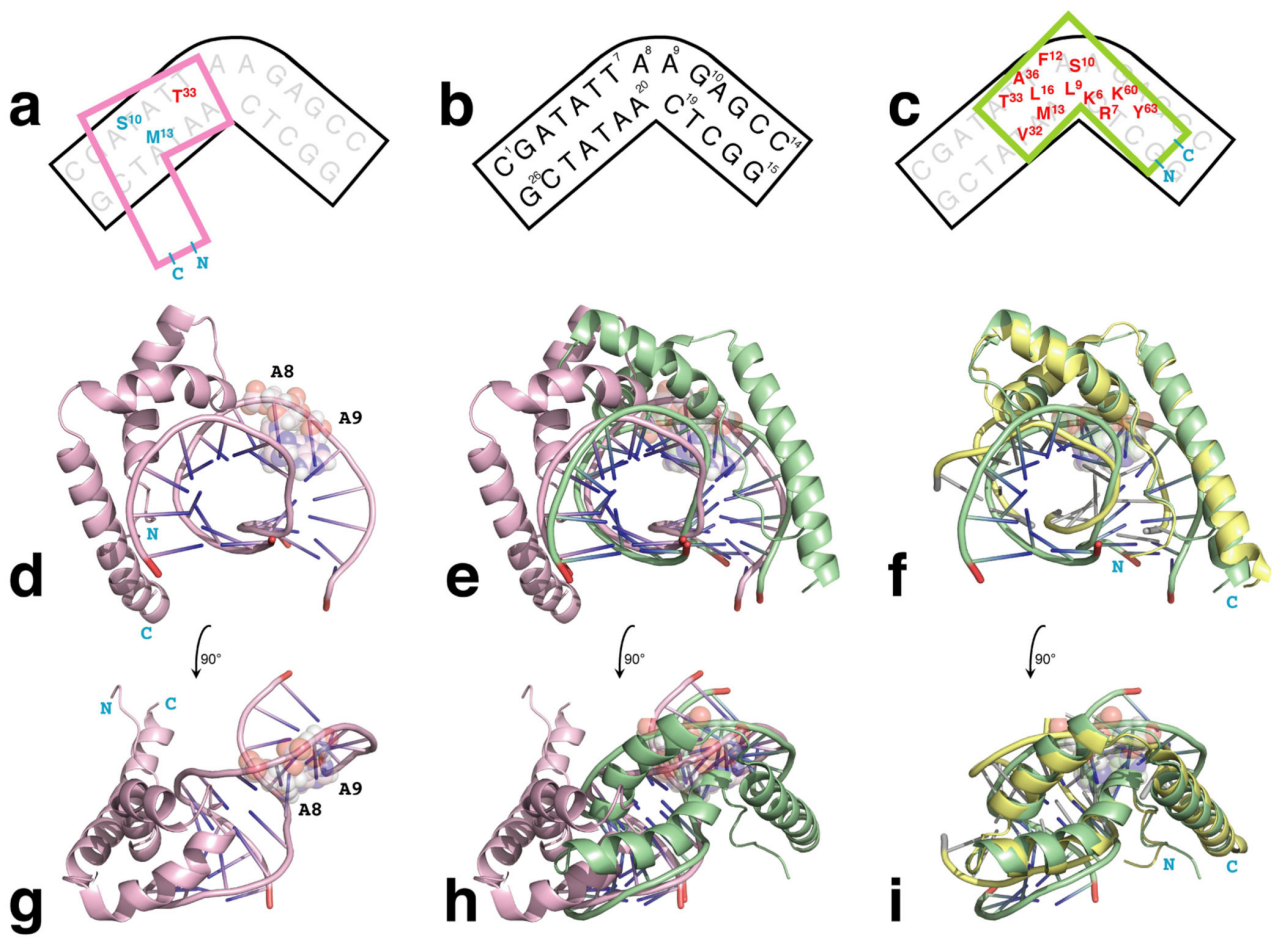
Relationship between the previously published structure of the HMG-D WT – DNA complex structure (PDB 1E7J),^[1]^ and the present structure of the HMG-D Y12F – DNA complex. a)–c) Schematic representations of a) PDB 1E7J, b) the 12:14 dA_2_ bulge DNA ligand used in both structures, and c) the HMG-D Y12F – DNA complex, showing the very different positioning of the protein in the two complex structures. Interfacial protein residues for which unambiguously assigned intermolecular NOEs were detected are shown in red; in a), residues for which other intermolecular restraints were defined in the original structure determination are shown in cyan. See text for further discussion. d) – i) Comparisons of the new structure of the HMG-D Y12F – DNA complex (shown in pale green), PDB 1E7J (shown in pink), and the X-ray structure of HMG-D bound to a canonical DNA ligand (PDB 1QRV, shown in yellow; only the DNA-bound copy of the protein molecule is shown). The two NMR structures were superposed using all atoms of the DNA ligand, while the new NMR structure and the X-ray structure of HMG-D were superposed using the backbone atoms of the protein (residues 5–70). Views in d), e) and f) are identical to one another and are each related to the views in g), h) and i) respectively by a 90° rotation about a horizontal axis. Nucleotides of the dA_2_ bulge are labelled in views d) and g). These superpositions show the very different positions of the protein on the DNA in the new and old structures.

Strikingly, this new structure shows a very different positioning and orientation of the protein on the DNA from that in the previously published model (Figure 4). In both cases the protein binds in the DNA minor groove as seen in other HMG-box protein DNA complexes,^[10,11b,15a,16,17,20]^ but in the new structure the protein directly covers the bent portion of the DNA containing the dA_2_ bulge, whereas in the previous model the protein binds to one side of the bulge and with the opposite orientation on the DNA. In the new structure, which, interestingly, is consistent with a prediction by Lippard for the binding of HMGB1 to cis-platinated DNA,^[21]^ the mode of binding is directly evidenced by an abundance of intermolecular NOE data for the key interfacial residues, as summarised in Figures 3b and 4c, and Supp. Table S2. In contrast, in the original work very few intermolecular NOE interactions could be detected or assigned, and in light of the present results one must consider the possibility that the previous model may be incorrect. Notably, the present study benefitted from the use of more modern NMR hardware (e. g. a cryogenically cooled probe) than in the original work, so although the newer data for the WT complex is still very markedly inferior to that for the Y12F complex and is still insufficient to allow an independent structure determination, it is now possible to propose tentative assignments for a handful of additional intermolecular cross-peaks for the WT complex, using a combination of the original assignment data (BMRB 4733) and analogy with the Y12F complex structure (see Supp. Figure S4a and Supp. Tables S3 and S4). Using these tentative new assignments to define intermolecular structural restraints allows calculation of a speculative model for the WT complex (Supp. Figure S4b), which, together with chemical shift perturbation data (Supp. Figures S5 and S6), suggests the WT protein has the same orientation on the DNA as does the Y12F mutant (i. e. opposite to that in PDB 1E7 J), but adopts a somewhat different, shifted pose within the minor groove. Such a structural shift is perhaps suggestive of translocations that might occur during 1D-diffusion of a non-specific HMG protein on DNA^[13,22]^; indeed, as mentioned earlier, if associated with breaking and reforming H bonds from Tyr12 OH to acceptors on the DNA on a millisecond timescale, it is possible that such translocation might be the process responsible for line-broad-ening of interfacial NMR signals in the WT complex. To take this speculation still further, one might imagine that the occurrence of such intermolecular H-bonds could represent a mechanism for controlling the rate of translocation of non-sequence-specific HMG proteins on DNA; this would be consistent with the observation that sequence-specific HMG proteins, where by definition translocation scarcely occurs and consequently there is no functional requirement to control its rate, frequently have phenylalanine residues at the position corresponding to Tyr12 of HMG-D (see Figure 1a).

The orientation of the protein on the DNA depicted in PDB 1E7 J (see Figure 4) probably arose chiefly due to an NOE tentatively assigned as linking the H^β^ protons of Ser10 to Thy4 H4’ in the DNA in the original study, which would be inconsistent with the new structure and was not seen in any of our subsequent repeat experiments with either the Y12F or WT complexes. In reality this may have been due to a mis-assigned intramolecular NOE between Ser ^13^C^β^H_2_ and Ser ^12^C^α^H signals, present due to incomplete isotopic labelling of the protein in the earlier protein preparations (Ser10 H^α^ and Thy4 H4’ had identical chemical shifts in the original WT spectra); Stott *et al*. have pointed out the hazard of misinterpreting such NOE cross peaks.^[17]^ Against these arguments, however, the new model is a significantly poorer fit to hydroxy-radical footprinting^[23]^ and FRET^[24]^ results previously reported for HMG-D WT binding a different dA_2_ bulge. This could perhaps be due to differences in experimental design, particularly in the DNA sequences used, that may have led to different binding arrangements; the significantly longer stems in the DNA ligands used in these other experiments allow for interactions with the disordered protein tail that cannot occur with the DNA ligand used for our NMR experiments.

In ascribing the improvement of data quality from the Y12F mutant to the elimination of intermediate-rate conformational dynamics within the protein-DNA interface, one key assumption is that the WT and Y12F proteins do not differ appreciably in their binding affinities for the dA_2_ bulge DNA ligand. We used isothermal calorimetry (ITC) to investigate the thermodynamics of binding to a closely related DNA ligand (this incorporated slightly extended stems so as to facilitate the ITC measurements as well as comparison with related literature data^[25]^). The results (Supp. Figure S7) show that the binding of either WT or Y12F HMG-D proteins to this dA_2_ bulge DNA show almost identical ΔG values of −11.2 kcalmol^-1^ (WT) or −11.0 kcal mol^−1^(Y12F); for the Y12F mutant, a small reduction in the enthalpic contribution is almost exactly balanced by a small increase in the entropic contribution, perhaps suggesting that loss of the intermolecular hydrogen bond to Tyr12 is at least partially compensated by increased motional freedom of the Phe12 sidechain in the mutant complex. The stoichiometry (2 proteins binding) is consistent with previous data for HMG-D binding to a linear DNA.^[11b]^

## Conclusions

In some respects this was a favourable case for application of the approach described here, given the extent of prior knowledge about the system. However, it is completely clear that the Y12F mutation brought a decisive improvement in the NMR properties of the complex, without which determination of the structure to high resolution would not have been possible. Intermolecular hydrogen bonds to tyrosine sidechain OH groups are quite common in protein-DNA and -RNA complexes,^[26]^ and substitution by phenylalanine may well be helpful for NMR studies in such cases if linewidths of interfacial signals are problematic in the wild-type complex. There has been at least one related previous example where a mutation improved the NMR properties of a protein-DNA complex, in that case by changing an aromatic protein residue to an aliphatic (Phe to Leu).^[27]^ More generally, we believe our work helps to demonstrate that, in principle, interfacial mutations may be capable of providing a useful way to alter the exchange behaviour, and hence potentially improve the spectral properties, of other types of complexes. Provided that the perturbation to the interface is sufficiently minor that the structure is still relevant, this could offer a useful addition to the armoury of techniques available for tackling difficult cases.

## Supplementary Material

Supporting Information

## Figures and Tables

**Table 1:** Structural statistics for the deposited ensemble of the complex of HMG-D Y12F bound to 14–12 dA_2_ bulge DNA

Structural Restraints	Protein	DNA	
NOE-based dist. restraints			
Intraresidue	366		157
Sequential	267		108
Medium (2 ≤ |i-j| ≤4)	214		
Long (|i-j| > 4)	136		
Cross-strand			13
Intermolecular		65	
Total	983		278
Hydrogen bond restraints	34 (17 H-bonds)		36 (6 G–C bp) 24 (6 A–T bp)
P–P dist. lower limit^[a]^			47
Torsion angle^[a]^			231
Basepair planarity^[a]^			10
**Stats.for accepted structures**			
Number of structs.		20 (of 60)	
Mean AMBER energies (kcal mol^−1^ ± S.D.)			
E(total)		−10708.5 ± 19	
E(van der Waals)		−1046 ± 7.9	
E(dist. restraints)		35.6 ± 7.4	
Dist. restraint viols. > 0.2 Å (av. # per struct.)		6.5 ± 1.6	
Angle restraint viols. > 5° (av. # per struct.)		8.2 ±2.4	
RMSD from ideal geom. used within AMBER			
Bond lengths		0.0101 Å	
Bond angles		2.00°	
**Ramachandran stats.**	**Res. 5–70**	**Res. 2–74**	
Most favoured	90.8%	90.0 %	
Additionally allowed	8.6%	9.1 %	
Generously allowed	0.2 %	0.4%	
Disallowed	0.5 %	0.5 %	
**Average atomic RMSD from av. struct. (± S.D.)**			
**5–70, 4–13, 16–23^[b]^**	**Protein**	**Complex**	**DNA**
(backbone)^[c]^	0.39 ± 0.11 Å	0.51 ± 0.13 Å	0.53 ± 0.16 Å
(all heavy)	0.96 ± 0.08 Å	0.88 ± 0.09 Å	0.65 ± 0.12 Å
**2–74, 1–14, 15–26^[d]^**	**Protein**	**Complex**	**DNA**
(backbone)^[c]^	0.70 ± 0.30 Å	0.91 ± 0.22 Å	0.85 ± 0.28 Å
(all heavy)	1.15 ± 0.22 Å	1.12 ± 0.18 Å	0.88 ± 0.23 Å

[a] Loose, non-experimental restraints to aid convergence for the DNA; see supplementary information experimental section for details.

[b] Residues 5–70 of protein; bases 4–13 and 16–23 of DNA.

[c] N, C^α^ and C’ atoms of protein; P, O5’, C5’, C4’, O4’, C3’, O3’, C2’, C1’ atoms of DNA.

[d] Residues 2–74 of protein; bases 1–14 and 15–26 of DNA.

## Data Availability

Structural co-ordinates and restraints for the complex of HMG-D Y12F bound to the 14:12 dA2 DNA bulge ligand have been deposited with the PDB database under accession code 8R1X. NMR chemical shift assignments have been deposited at the BMRB under accession code 34874 for the complex and 52208 for the free protein backbone.
