# Supplementary material for: A Single Interfacial Point Mutation Rescues Solution Structure Determination of the Complex of HMG-D with a DNA Bulge
Source: Chembiochem. Author manuscript; Available in PMC 2025 Feb 25. (PMC7617430; doi:10.1002/cbic.202400395)
Supplement: Supporting Information [file EMS203401-supplement-Supporting_Information.pdf]

ChemBioChem

Supporting Information

A Single Interfacial Point Mutation Rescues Solution Structure Determination of the Complex of HMG-D with a DNA Bulge

Guy R. Hill, Ji-Chun Yang, Laura E. Easton, Rachel Cerdan, Stephen H. McLaughlin, Katherine Stott, Andrew A. Travers, and David Neuhaus*

Supporting Information

A Single Interfacial Point Mutation Rescues Solution Structure Determination of the Complex of HMG-D with a DNA Bulge.

Guy R. Hill‡, Ji-Chun Yang‡, Laura E. Easton, Rachel Cerdan, Stephen H. McLaughlin, Katherine Stott, Andrew A. Travers, and David Neuhaus*.

MRC Laboratory of Molecular Biology, Francis Crick Avenue, Cambridge CB2 0QH, U.K.
LPHI, Univ. Montpellier, CNRS, Inserm, Place Eugène Bataillon, 34095, Montpellier, France.
Department of Biochemistry, University of Cambridge, 80 Tennis Court Road, Cambridge CB2 1GA, U.K.

‡ These two authors contributed equally to the project

* To whom correspondence should be addressed. Email: dn@mrc-lmb.cam.ac.uk

Table of Contents

Experimental Section

Figure S1 1D NMR spectra of the complexes of HMG-D Y12F and HMG-D WT with the dA_2 bulge DNA ligand.

Figure S2 [^{15}N,^{1}H] HSQC spectra of the complexes of HMG-D Y12F and HMG-D WT with the dA_2 bulge DNA ligand.

Figure S3 Rmsd profile for the final ensemble of 20 structures of the complex of HMG-D Y12F mutant with the dA_2 bulge DNA ligand.

Figure S4 Speculative model of the complex of HMG-D WT with the 12:14 dA_2 DNA bulge ligand.

Figure S5 Chemical shift perturbations (CSPs) for the DNA ligand.

Figure S6 Chemical shift perturbations (CSPs) for the protein.

Figure S7 ITC binding isotherms for HMG-D Y12F and HMG-D WT titrated into DNA.

Table S1 Numbers of intermolecular NOE-based distance restraints reported in different structure determinations of HMG box protein complexes with DNA.

Table S2 Intermolecular NOE-based distance restraints used in the calculation of the NMR structural ensemble of HMG-D Y12F bound to 14-12 dA_2 bulge DNA.

Table S3 Putative assignments for intermolecular NOE cross-peaks in the filtered NOESY spectrum of the complex of HMG-D WT bound to 14-12 dA_2 bulge DNA.

Table S4 DNA protons in structure PDB 1E7J that lie within 5 Å of protein methyl groups assigned as showing intermolecular NOE cross-peaks for the complex of HMG-D WT bound to 14-12 dA_2 bulge DNA.

Separate file: Co-ordinate file (in mmCIF format) for the model of the complex of HMG-D WT with the dA_2 bulge DNA as shown in Figure S4b.

EXPERIMENTAL SECTION

Gene Cloning

The full-length HMG-D wild-type (WT) sequence (residues 1-112) was subcloned into the NdeI/XhoI sites of the pET13a expression vector. The Y12F mutation was carried out using the QuikChange protocol (Stratagene).

Protein expression and purification

Full-length HMG-D WT and Y12F mutant were expressed using *E. coli* strain BL21 (DE3) cells. Unlabelled protein was expressed in LB medium at 37 °C to 0.8-1.2 A_{600} before inducing with IPTG (0.5 mM) and then incubating at 25 °C for 4 hours, followed by harvesting at 4 °C. Protein samples uniformly labelled with ^{15}N and ^{13}C were expressed in M9 minimal media, initially overnight at 37 °C without carbon or nitrogen sources to A_{600} approx. 1.5, then following addition of $^{15}NH_4Cl$ (1 g/L) and $^{13}C_6$-glucose (2 g/L) for 3 hours at 25 °C, before inducing with IPTG (1 mM) and incubating at 25 °C for 4 hours, followed by harvesting at 4 °C.

For samples for NMR, purification of the protein was essentially as previously described.[1] All steps were performed at 4 °C or on ice. Cells were resuspended in buffer A (50 mM HEPES pH 8, 500 mM NaCl, 1 mM EDTA, 1 mM DTT, Roche Complete Protease Inhibitor Cocktail EDTA free; 1 tablet per 50 ml) and lysed by sonication, the lysate cleared by centrifugation (18,000 rpm for 15 min at 4 °C) and dialysed against buffer B (as buffer A except NaCl concentration 50 mM). Initial purification was achieved using fractional precipitation with $(NH_4)_2SO_4$; finely ground $(NH_4)_2SO_4$ was slowly added during 15 min to the lysate at 4 °C to reach 65% saturation (45.5 g per 100 ml) while limiting pH rises by small additions of dilute HCl; the precipitate was then removed by centrifugation (15,000 rpm for 20 min at 4 °C) and discarded; a further precipitate, containing mostly HMG-D protein, was induced by adding more $(NH_4)_2SO_4$ to the lysate to reach 95% saturation (63 g per 100 ml) and stirring for 30 min at 4 °C. This second precipitate was collected by centrifugation (15,000 rpm for 20 min at 4 °C), then resuspended in buffer B and $(NH_4)_2SO_4$ removed by dialysis against the same buffer. Further purification was achieved by column chromatography, first using a DEAE Sephacel column (Pharmacia) to remove anionic impurities, then with a source S size exclusion column (Pharmacia) eluted with a gradient of 50 mM-1 M NaCl (other components remaining constant as for buffer B). The pooled HMG-D fractions were transferred to NMR buffer (10 mM NaH_2PO_4 pH 6.0, 20 mM NaCl and 0.02% (w/v) NaN_3) and concentrated to 1-2 mM using a Vivaspin column (20 ml, 10 kDa cutoff), and final protein concentration estimated by UV spectroscopy.

For samples for Isothermal titration calorimetry (ITC), a simpler purification protocol was used that gave essentially identical results. All steps were performed at 4 °C or on ice. Cells were resuspended in buffer C (50 mM HEPES, pH 8, 50 mM NaCl, 1 mM EDTA, 1 mM PMSF, 0.5 mM TCEP, 0.1% (v/v) Triton-X100 and Roche Complete Protease Inhibitor Cocktail EDTA free; 1 tablet per 50 ml) and lysed by sonication. The lysate was cleared by centrifugation (19,500 rpm at 4 °C), then loaded on a 5 ml HiTrap SP-HP FF column (GE Healthcare) equilibrated in buffer D (50 mM HEPES, pH 8, 50 mM NaCl, 1 mM EDTA, 0.5 mM TCEP). The column was washed with buffer D and the proteins eluted over a gradient from 50 mM to 1 M NaCl, then dialysed in a 3.5 kDa cutoff snakeskin membrane against buffer D at 4 °C overnight. Proteins were then loaded onto a 5 ml HiTrap Heparin HP column (GE Healthcare) equilibrated with buffer D, washed with a gradient of 50 –

200 mM NaCl and eluted with a gradient of 200-700 mM NaCl, after which they were passed over a Superose S75 column (GE Healthcare) in buffer D. Finally, the eluted proteins were dialysed in a 3.5 kDa cutoff snakeskin membrane against ITC buffer (10 mM sodium phosphate, pH 6, 20 mM NaCl) at 4 °C overnight.

Preparation of bulged dsDNA ligands

DNA oligos with the following sequences were either synthesized in-house (14- and 12-mers for NMR) or purchased from Integrated DNA technologies (22- and 20-mers for ITC) (the underlined nucleotides denote the bulged A's):

GCATATT<u>AA</u>GAGCC

GGCTCAATATCG

GTCACGATATT<u>AA</u>GAGCCATGC

GCATGGCTCAATATCGTGAC

These were further purified either by reverse-phase HPLC (for the NMR samples) or preparative PAGE electrophoresis (for the ITC samples; these were run on an 8 M urea, 12% (w/v) polyacrylamide gel at 30 W for 6 hours, followed by excision from the gel and extraction by electroelution), then either lyophilized and resuspended in H_2O or ^2H_2O (NMR samples) or buffer exchanged into ITC buffer (ITC samples), and lastly the 14-mer and 12-mer (for NMR) or the 22-mer and 20-mer (for ITC) were annealed at a 1:1 ratio at 90 °C followed by slow cooling to form duplex. Correct formation of the bulged dsDNA was verified either by ^1H NMR (NMR samples) or native PAGE (ITC samples).

Preparation of protein:DNA complex for NMR

NMR samples of the complex of either full-length HMG-D Y12F or full-length HMG-D WT with the 14:12 dA_2 bulge ligand were formed by adding an equimolar amount of [^{15}N,^{13}C] labelled protein solution in NMR buffer to a DNA solution in NMR buffer that had been concentrated to 10 mM to minimise dilution of protein on mixing. To establish that the protein was fully in the bound state, the backbone NH signal of residue Glu34 was monitored, as this signal was well-resolved in the [^{15}N,^1H] HSQC spectrum and showed particularly large changes on binding. Samples of the complexes in ^2H_2O were prepared from H_2O samples by repeated cycles of dilution with deuterated NMR buffer followed by careful partial concentration using a Vivaspin (20 ml, 10 kDa cutoff) centrifugation column.

NMR spectroscopy

All NMR measurements employed in-house Bruker Avance I 500 MHz or Avance I or Avance III HD 800 MHz spectrometers equipped with 5 mm [^1H,^{13}C,^{15}N]-cryogenic probes. NMR samples were prepared in NMR buffer with 5% added ^2H_2O. Experiments were conducted at 27 °C unless otherwise stated, and ^1H chemical shifts were calibrated using sodium 3,3,3-trimethylsilylpropionate (TSP) as an external ^1H reference; ^{15}N and ^{13}C chemical shifts were indirectly referenced to the ^1H shifts using the ratio of gyromagnetic ratios.[2] Essentially complete resonance assignments were obtained for the protein and DNA (excepting the H5'/H5" protons) in the ordered part of the complex, together with partial backbone assignments for the disordered tail and (separately) for the free

protein, using the experiments described below; assignments for the DNA additionally made use of the data in BMRB 4734.

For free HMG-D Y12F protein in H_2O solution the following datasets were acquired: 2D datasets: [^{15}N-^{1}H] HSQC; [^{13}C-^{1}H] HSQC. 3D datasets: CBCANH; CBCA(CO)NH.

For HMG-D Y12F protein complexed to 14:12 DNA in H_2O solution the following datasets were acquired: 2D datasets: [^{15}N-^{1}H] HSQC; [^{13}C-^{1}H] HSQC (also run for the complex of the WT protein); [^{1}H-^{1}H] NOESY (τ_m = 60 ms) ; [^{1}H-^{1}H] NOESY (τ_m = 150 ms). 3D datasets: HNCA; HN(CO)CA; CBCANH; CBCA(CO)NH; HBHANH; HBHA(CO)NH; [^{1}H-^{13}C-^{1}H] HCCH-COSY; [^{1}H-^{13}C-^{1}H] HCCH-TOCSY; ^{15}N NOESY-HSQC (τ_m = 150 ms); ^{13}C NOESY-HSQC (τ_m = 150 ms; separate experiments acquired for aliphatic and aromatic regions in ^{13}C).

For HMG-D Y12F protein complexed to 14:12 DNA in $^{2}H_2O$ solution the following datasets were acquired: 2D datasets: [^{13}C,^{15}N]-filtered [^{1}H-^{1}H] NOESY (τ_m = 100 ms, with filter elements set to reject ^{13}C and ^{15}N coupled signals in F_1 and to accept only ^{13}C coupled signals in F_2); [^{13}C,^{15}N]-filtered [^{1}H-^{1}H] NOESY (τ_m = 200 ms, with filter elements set to reject ^{13}C and ^{15}N coupled signals in F_1 and to accept only ^{13}C coupled signals in F_2); identical versions of these 2D filtered NOESY experiments were also run with a sample of the complex of the WT protein in $^{2}H_2O$. 3D datasets: ^{13}C-filtered ^{13}C NOESY-HSQC (τ_m = 150 ms, with filter elements set to reject ^{13}C- and ^{15}N-coupled (protein) signals in F_1). The filters to reject ^{15}N-coupled protons in these experiments served to suppress signals from slowly exchanging amide NH signals that persisted in these samples in $^{2}H_2O$ solution.

NMR data were processed using the program TopSpin (versions 3.2 and 3.5; Bruker BioSpin GmbH), and analysed using the program Sparky (version 3.115).[3] Contour levels used in the spectra shown in Figure 1 were normalised using intensity measurements for signals expected to be unaffected by the Y12F mutation. For the [^{13}C, ^{1}H] HSQC spectra intensity matching of the I30 $C^{\delta1}/H^{\delta1}$ cross peak was used, but for the filtered NOESY spectra no suitable cross peaks were available in the displayed spectra, so instead intensity comparison of the overall protein signal envelope as seen in 1D spectra recorded under identical conditions for the two samples was used to determine the concentration ratio, which was found to be 1:1.87 (WT:Y12F) (see Supp. Figure S1; in order to keep the signal-to-noise ratio comparable between the two filtered NOESY spectra, the number of transients collected for the WT sample was set to be $(1.87)^2$ = 3.5 times higher than that for the Y12F sample).

NMR structure calculations

Distance restraints were derived from assigned cross peaks measured in the NOESY spectra listed above. Intra-protein cross peaks were classified into semi-quantitative distance categories based on intensities of cross peaks observed for protons in α-helices ($d_{NN}(i,i+1)$ = 2.9 Å, $d_{N\alpha}(i,i+3)$ = 3.5 Å) as follows: very strong, d <= 2.3 Å (13 restraints); strong, d <= 2.9 Å (162 restraints), medium, d <= 3.5 Å (227 restraints); weak, d <= 5.0 Å (581 restraints). Lower bounds for all NOE restraints were set to zero,[4] and no multiplicity corrections were required since r^{6} summation was used for restraints involving groups of equivalent or non-stereoassigned spins.[5] This distance classification was extended to include intermolecular and intra-DNA restraints by using intensities observed for such cross peaks in cases where they were well-resolved in spectra that also included intra-protein cross peaks (in the case of the intra-DNA cross peaks, a further category of very weak (d <= 6.0 Å) was

also defined). For the intra-DNA cross peaks this process yielded 4 restraints in the strong category, 63 in the medium, 202 in the weak and 9 in the very weak; for the intermolecular cross peaks, it yielded 9 restraints in the strong category, 24 in the medium and 32 in the weak (of which 2 were defined ambiguously). Hydrogen bonding restraints ($d_{O \leftarrow HN}$ <=2.3 Å and 0.5 Å <= $d_{O \leftarrow N}$ <=3.3 Å) were defined for those residues where NH amide signals could be still observed several hours after transferring the sample into ^2H_2O; since it was clear from preliminary structure calculations that all of these residues were within α-helices, in each case the acceptor atom was set to be the oxygen atom of the *i-4* backbone carbonyl group.

A number of non-experimental restraints were also applied to improve convergence of the DNA by restricting the search to reasonable regions of conformation space. These restraints were slightly modified from those used in the earlier study[6] and comprised the following: i) H-bond restraints for Watson-Crick basepairs (for G-C pairs these were set to N1-N3 < 3.0 Å, H1-N3 < 2.0 Å, N2-O2 < 3.0 Å, H21-O2 < 2.0 Å, O6-N4 < 3.0 Å, O6-H41 < 2.0 Å; for A-T pairs they were set to N1-N3 < 3.0 Å, N1-H3 < 2.0 Å, N6-O4 < 3.0 Å, H61-O4 < 2.0 Å); ii) loose torsion angle restraints to maintain very approximate B-form geometry in the DNA stems, excepting T7 and A20 (these were set to $-110° \leq \alpha \leq -30°$, $+145° \leq \beta \leq +195°$, $+35° \leq \gamma \leq +85°$, $+150° \leq \delta \leq +70°$, $-210° \leq \varepsilon \leq -130°$, and $-160° \leq \zeta \leq -60°$); iii) loose torsion angle restraints to maintain an approximate 2'-endo puckered conformation in the ribose rings within the stems, excepting T7 and A20 (these were set to $-40° \leq \nu_0 \leq 0°$, $+10° \leq \nu_1 \leq +50°$, $-50° \leq \nu_2 \leq -10°$; $0° \leq \nu_3 \leq +40°$, and $-20° \leq \nu_4 \leq +20°$); iv) loose basepair planarity restraints for basepairs in the stems, excepting T7-A20 and G10-C19; v) minimum distances between backbone phosphorous atoms to prevent excessive kinking (these were applied only during stage 2 of the XPLOR-NIH calculations and during the AMBER 11 calculations (see below), and were set to P-P < 10.5 Å for the 1-3 related pairs G2-T4, A3-A5, T4-T6, A5-T7, G10-G12, A11-C13, G12-C14, G16-T18, C17-C19, A20-T22, A21-A23, T22-T24, A23-C25 and T24-G26; P-P < 14.0 Å for the 1-4 related pairs G2-A5, A3-T6, T4-T7, G10-C13, A11-C14, G16-C19, A20-A23, A21-T24, T22-C25 and A23-G26; and P-P < 17.0 Å for the 1-5 related pairs G2-T6, A3-T7, G10-C14, A20-T24, A21-C25 and T22-G26), or to prevent unreasonably close proximity of the stems to one another (these were set to P-P < 9.0 Å for the inter-stem pairs A3-T18, A3-C19, A3-A20, T4-T18, T4-C19 and T4-A20), or to prevent cross-strand distances becoming too short (these were set to P-P < 16.0 Å for the cross-strand distances G2-G26, A3-C25, T4-T24, A5-A23, T6-T22, T7-A21, G10-A20, A11-C19, G12-T18, C13-C17 and C14-G16). In all cases, these minimum phosphorous-phosphorous distance restraints corresponded to significantly shorter distances than the corresponding minimum P-P distances seen in the very highly kinked DNA double helix of the TATA-box protein-DNA complex (PDB 1vtl).[7]

Initial structures (60) were calculated using a two-stage simulated annealing protocol in the program XPLOR-NIH 2.28,[8] as previously described by Cerdan et al.[6] For the calculations, the protein chain was limited to residues 2-74; residues 75-112 are known to be disordered in both the free and bound states,[6] and in the native protein residue Met1 is removed enzymatically in vivo,[1a] even though in overexpressed protein as prepared for NMR incomplete processing can result in variable amounts of

protein containing Met1 persisting (as evidenced in Figure 1).[9] Since the XPLOR-NIH calculations employed r^{6} summation for all groups of equivalent protons and non-stereospecifically assigned prochiral groups, and since no stereoassignments were made (and the assignment-swapping protocol within XPLOR-NIH for deriving stereoassignments indirectly during the structure calculation itself was not applied), all distance restraints involving protons within such groups were converted to group restraints (by using wildcards such as Hβ*). All lower bounds were set to zero.[4] Starting structures were constructed by fully randomizing all rotatable backbone angles in the protein and both DNA chains and placing the three chains with their centres of mass at the vertices of an equilateral triangle with sides of length 60 Å. During the first part of the high-temperature dynamics stage, the three chains were constrained to these initial starting positions by fixing the coordinates of atoms Ala36 C^{α}, T7 C1', and A20 C1'; this was found empirically to improve convergence. Stage 1 of the XPLOR-NIH protocol thus began with Powell energy minimization (1000 steps) and 15,000 steps of Langevin dynamics at 1000 K during which the chain separations were constrained, followed by a further 30,000 steps of Langevin dynamics at 1000 K with the chain separations unconstrained, then increase of the van der Waals force constant and tilting of the NOE potential function asymptote in 3000 step cycles, switching to a square-well NOE function and cooling to 300 K in 2000 step cycles before a final Powell minimization (1500 steps). The force constants used for both the distance and the dihedral angle restraints were 50 kcal mol^{-1}. In stage 2 of the XPLOR-NIH protocol, structures were subjected to Powell energy minimization (1000 steps), Langevin dynamics at 1000 K while progressively increasing the torsion angle force constant in 2000 step cycles, switching to a square-well NOE function then cooling to 300 K in 600 step cycles and final Powell minimization (4000 steps).

The resulting 60 structures were then all subjected to a further stage of refinement using the program AMBER 11.[10] Restraints in XPLOR-NIH format were converted to AMBER format using a home-written script that, *inter alia*, added corrections to the NOE-derived upper bound distances to allow for signal multiplicities within equivalent and non-steroassigned groups; this was required because AMBER uses r^{6} averaging rather than r^{6} summation.[5b] Energy minimization (100 steps of steepest descent followed by 1900 steps of conjugate gradient minimization) was applied in a full force field and incorporating an implicit water-solvent model using the generalized Born method [11] (igb=1); the experimental distance restraints as well as the non-experimental distance, torsion angle, chirality and basepair planarity restraints were applied throughout, with force constants set to 20 kcal $mol^{-1}Å^{-2}$ for distance restraints, 20 kcal $mol^{-1}rad^{-2}$ for torsion angle restraints, except for ω torsion angle restraints that were set to 177° < ω < 183° with a force constant of 50 kcal $mol^{-1}rad^{-2}$; force constants were set to 10 kcal $mol^{-1}rad^{-2}$ for chirality restraints and 20 kcal $mol^{-1}rad^{-2}$ for base-pair planarity restraints. The model of the WT complex (Supp. Figure S4b) was calculated using the same protocol as above, except that the AMBER calculations were extended to include an annealing stage (5,000 steps of heating from 10 K to 500 K with time-constant (tautp) = 0.4, then 13,000 steps of cooling to 100 K with tautp = 4.0, 1,000 steps of cooling at 0 K with tautp = 1.0 and 1,000 steps of cooling at 0 K with tautp = 0.05, followed by repeating the whole annealing sequence) followed by a further energy minimisation stage (100 steps of steepest descent followed by 1900 steps of conjugate gradient minimization).

The 20 models with lowest total energy (from a total of 60) were accepted to form the final ensemble (Figure 3 and Supp. Figure S3). Ramachandran statistics were calculated using the program PROCHECK-NMR,[12] and the program CLUSTERPOSE[13] was used to calculate the mean rmsd of the ensemble to its mean structure. Structures were visualized using the program PYMOL.[14]

ITC measurements

ITC experiments were performed using an iTC200 titration calorimeter (Malvern Panalytical). Protein in ITC buffer at a concentration of 0.55 mM for wild-type and 0.51 mM for Y12F mutant was injected into the cell containing 20 μM DNA. The sequence of injections consisted of one 0.5 μl injection (discarded in the data analysis) followed by 19 injections of 2 μl each at 25 °C. Data acquisition was carried out with a reference power of 6 μcal/s, initial delay of 60 s with spacing between injections of 180 s. The heats of dilution were minimal for each protein. Each peak was integrated using Malvern Panalytical MicroCal PEAK PEAQ-ITC software 1.41 and raw data and fits were plotted in Prism 10.0.3 (GraphPad Software).

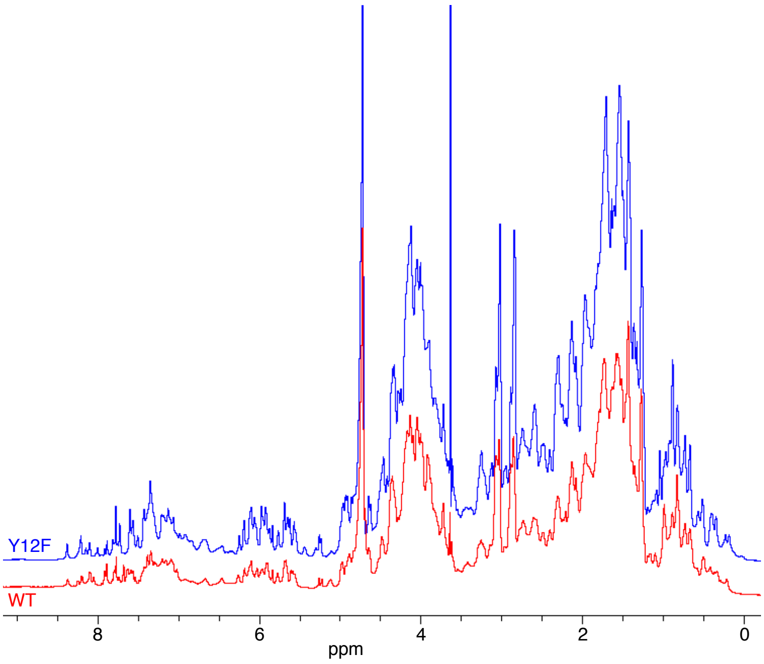

Figure S1: 1D NMR spectra of the complexes of HMG-D Y12F (blue) and HMG-D WT (red) with the dA_2 bulge DNA ligand.

The relative intensity of these two spectra establishes that the ratio of the concentration of the WT complex sample to that of the Y12F complex sample was 1:1.87. Spectra were recorded at 27 °C and 800 MHz.

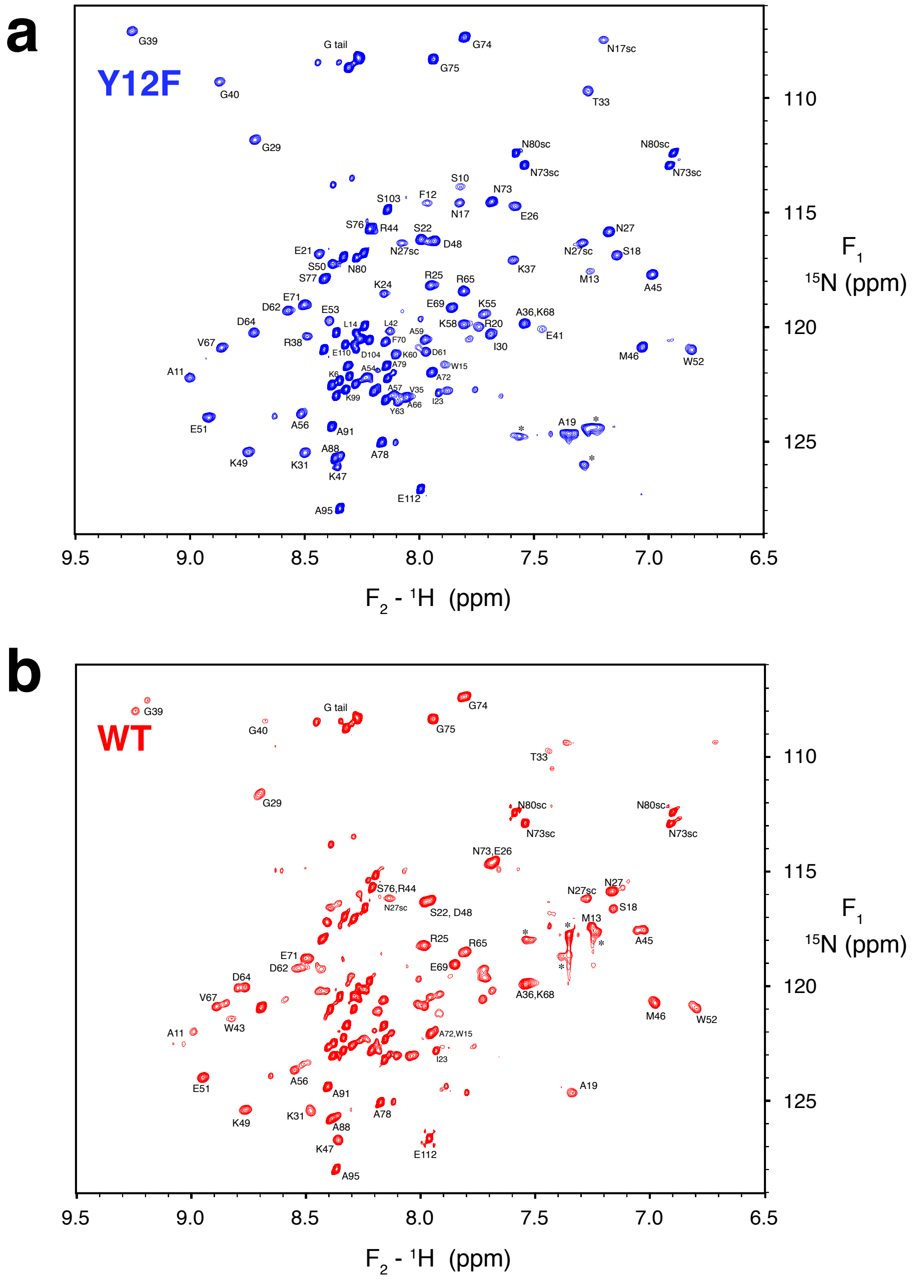

Figure S2: [^{15}N,^{1}H] HSQC spectra of the complexes of HMG-D Y12F and HMG-D WT with the dA₂ bulge DNA ligand.

a) Complex of HMG-D Y12F (blue), and b) complex of HMG-D WT(red); spectra were recorded at 27 °C and 800 MHz. In both spectra, signals from the disordered and highly repetitive C-terminal tail are expected in the crowded central part of the spectrum but were mostly not assigned individually. Peaks marked with an asterisk (*) are folded in the ^{15}N dimension and presumably arise from sidechain NH^{ε} signals of Arg residues. Assignments for the WT spectrum are based on BMRB 4734 (no assignments are indicated in the crowded central region of the WT spectrum due to the possibility of small shifts relative to the BMRB 4734 data). Doubling of some signals may be due to a slow conformational equilibrium within the complex, that occurs in addition to the intermediate rate process responsible for the signal loss discussed in the main paper; the Y12F mutation eliminates both these types of effects from the spectrum.

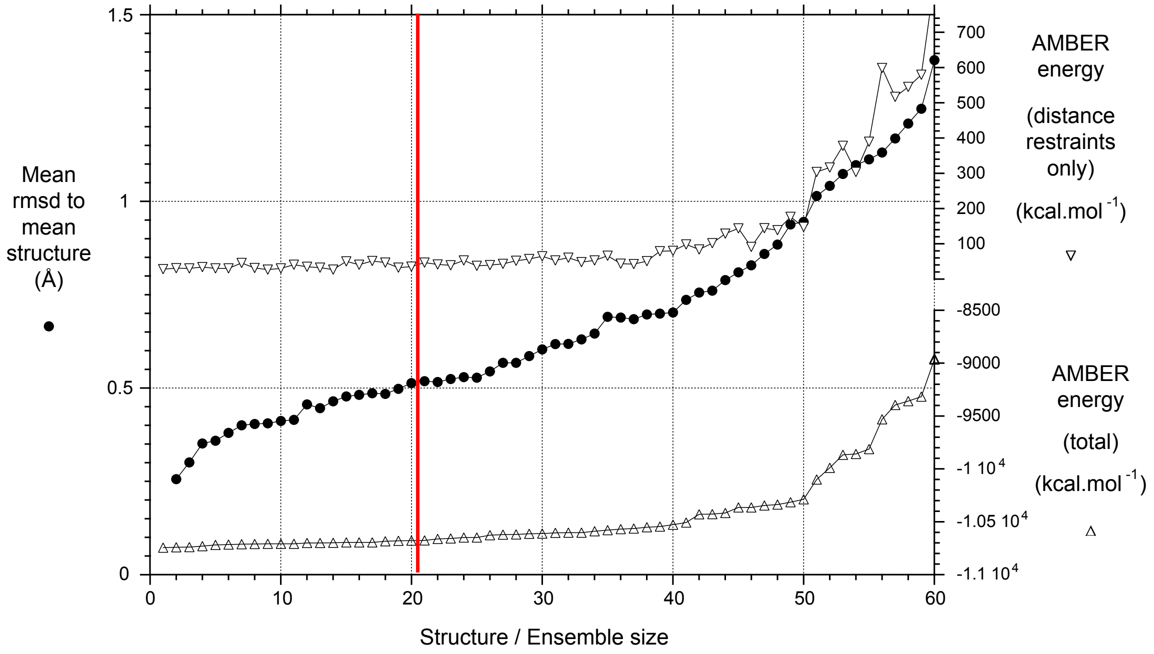

Figure S3: Rmsd profile for the final ensemble of 20 structures of the complex of HMG-D Y12F mutant with the dA_2 bulge DNA ligand.

A total of 60 structures were calculated. Rmsd values (filled circles) are independently calculated using each ensemble size, adding successive structures in order of increasing AMBER total energy term. Open triangles represent the AMBER energy terms as indicated. Only structures to the left of the vertical red line were included in the deposited co-ordinates and when calculating the structural statistics. Rmsd calculations employed the program CLUSTERPOSE.[13]

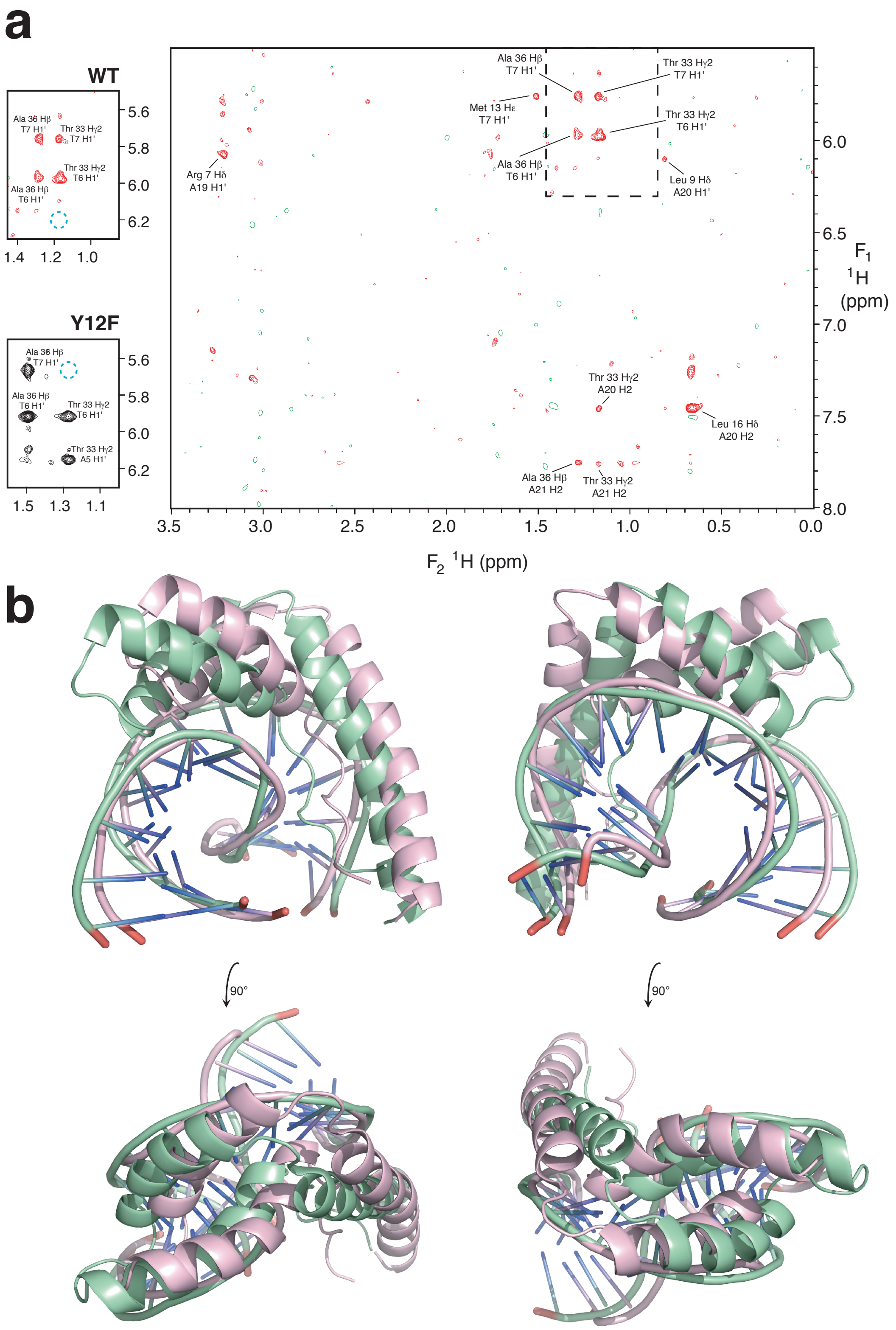

Figure S4: Speculative model of the complex of HMG-D WT with the 12:14 dA$_2$ DNA bulge ligand.

a) Part of the filtered NOESY spectrum (τ_m = 100 ms, chosen to minimise the possibility of spin-diffusion artefacts) of the complex of HMG-D WT with the 12:14 dA$_2$ DNA bulge ligand, recorded at 800 MHz and 27 °C (+ve contours plotted in red, -ve contours in green). The tentative intermolecular NOE cross-peak assignments indicated on the spectrum were derived using a combination of the

originally published assignments for the WT complex (BMRB 4734) and analogy with data for the complex of the Y12F mutant (this work); for further details of how these assignments were derived see Supp. Tables S3 and S4. The two insets show the region containing peaks from Thr33 Hγ2 and Ala36 Hβ to the H1' protons of A5, T6 and T7 (see box with dashed lines in the main figure); the upper inset is from the WT spectrum, the lower inset from the corresponding spectrum (τ_m = 100 ms) of the Y12F complex. The different patterns visible in the two insets clearly demonstrate that the pose of the protein in the minor groove of the DNA must differ between the WT and Y12F complexes (dashed cyan circles indicate positions where a prominent peak present in one spectrum is missing in the other).

b) Speculative model of the complex of HMG-D WT with the dA_2 bulge DNA (pink) superposed on the lowest energy structure of the complex of the Y12F mutant (light green). The model of the WT complex was calculated using the same protocol as for the deposited ensemble of the Y12F complex, except that the AMBER calculations were extended to include a long annealing stage (see Experimental Section for details; the WT ensemble member having the best fit to the DNA backbone of the Y12F complex lowest energy structure is shown). The only intermolecular restraints employed during these calculations were those corresponding to the 11 assigned cross-peaks shown in panel a, using upper distance limits specified in Supplementary Table S3. All other restraint files used for the calculation of the model were identical to those used for calculation of the complex of the Y12F mutant (modified by removal from the intra-protein restraints of the 7 restraints involving the Hζ proton of Phe12, and inclusion of four further P-P minimum distance restraints (r > 10.5 Å) in the bulge region of the DNA for the 1-3 related pairs T6-A8, T7-A9, A8-G10 and A9-A11). The two structures were superposed using the well-ordered region of the DNA ligand (backbone atoms of nucleotides 4-13 and 16-23, rmsd = 2.09 Å). It should be noted that this model cannot represent the dynamics that must be present within the WT complex, as evidenced by the extensive line-broadening referred to in the main text as well as the doubling of some interfacial signals such as many of the amide cross peaks seen in Supp. Figure S2b and for some methyl signals (e.g. Leu16 Hδb); given that the few intermolecular NOE cross-peaks detected are presumably amongst the most intense in the spectrum, we suggest this model should be interpreted as corresponding to the major form of the complex present in the sample. A co-ordinate file (in mmCIF format) for the model of the complex of HMG-D WT with the dA_2 bulge DNA is provided in the Supplemental Information.

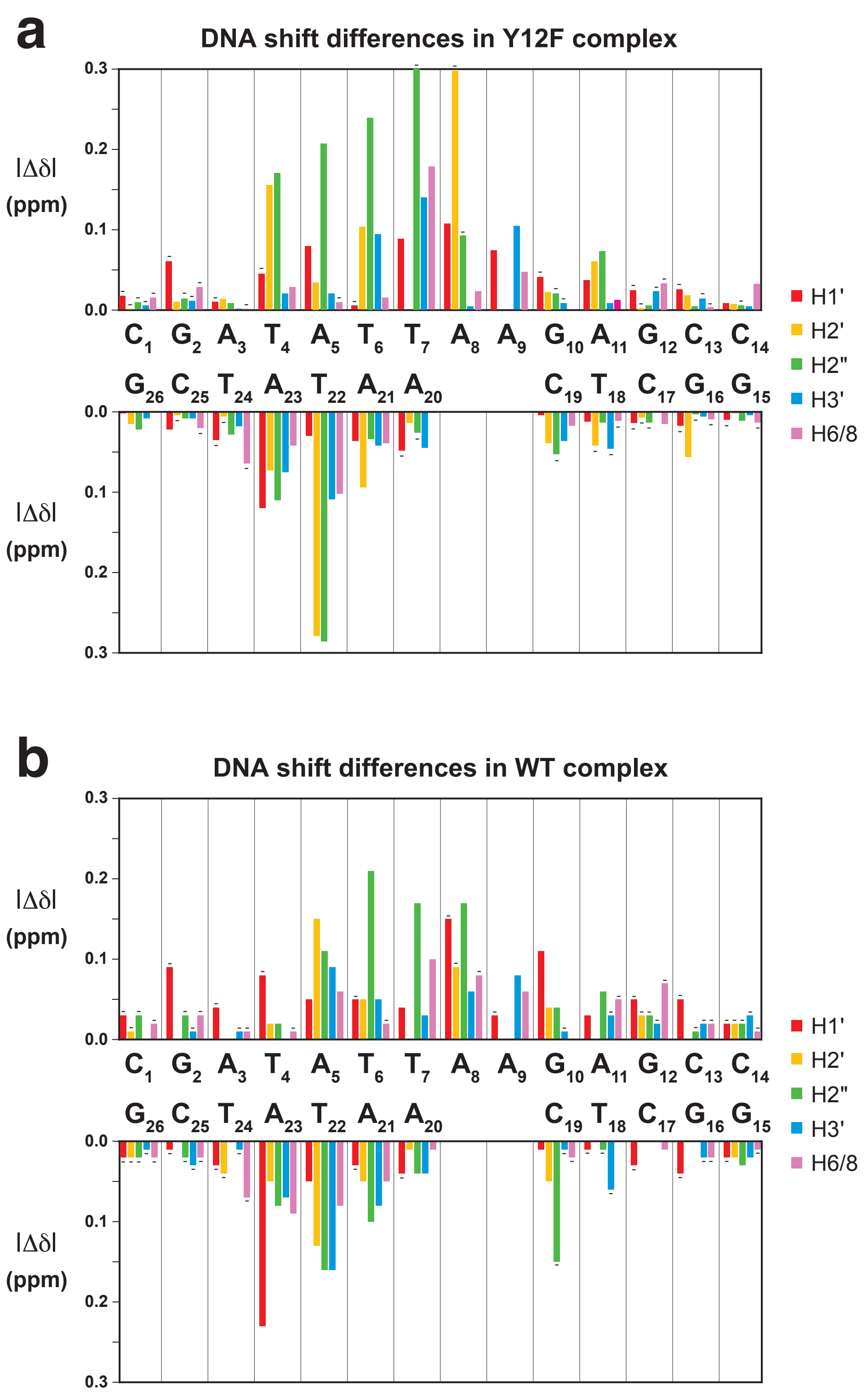

Figure S5: Chemical shift perturbations (CSPs) for the DNA ligand.

Differences of free and bound chemical shifts for ^{1}H resonances of the dA_2 bulge DNA upon binding a) full-length HMG-D Y12F and b) full-length HMG-D WT. Cases where the shift difference δ(free) – δ(bound) is negative are indicated with a minus sign above the relevant bar in the histogram. The value of $|\Delta\delta|$ for T_7 H2”, which is shown truncated in a), is -0.302ppm; values for δ(free) were taken from BMRB 4733.

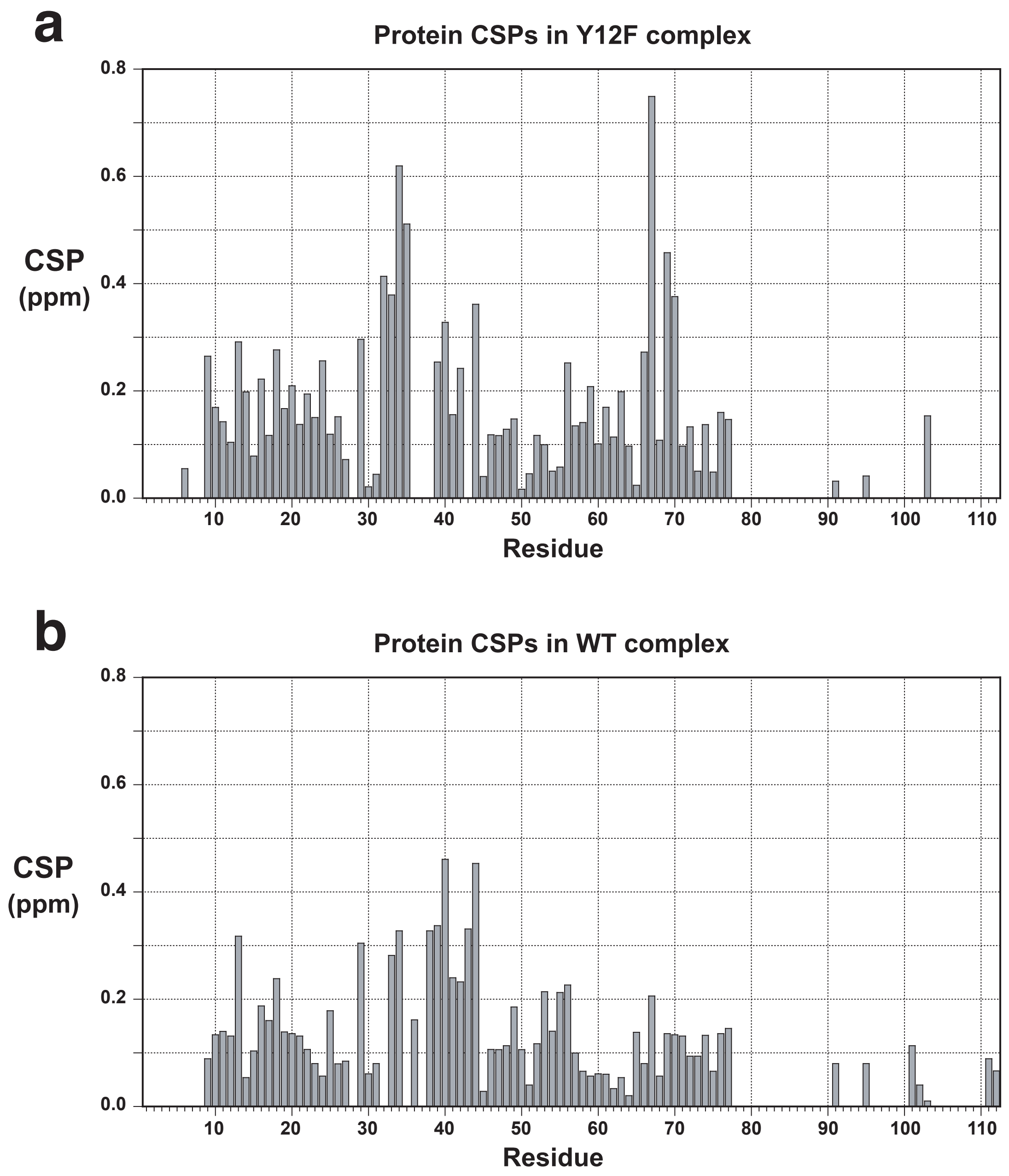

Figure S6: Chemical shift perturbations (CSPs) for the protein.
CSP values of backbone amide groups of a) full-length HMG-D Y12F and b) full-length HMG-D WT on binding to the dA_2 bulge DNA are shown, calculated as $((\Delta\delta^1H)^2+0.14(\Delta\delta^{15}N)^2)^{1/2}$.[20] Values for HMG-D WT were calculated using data from BMRB 4732 and 4734.

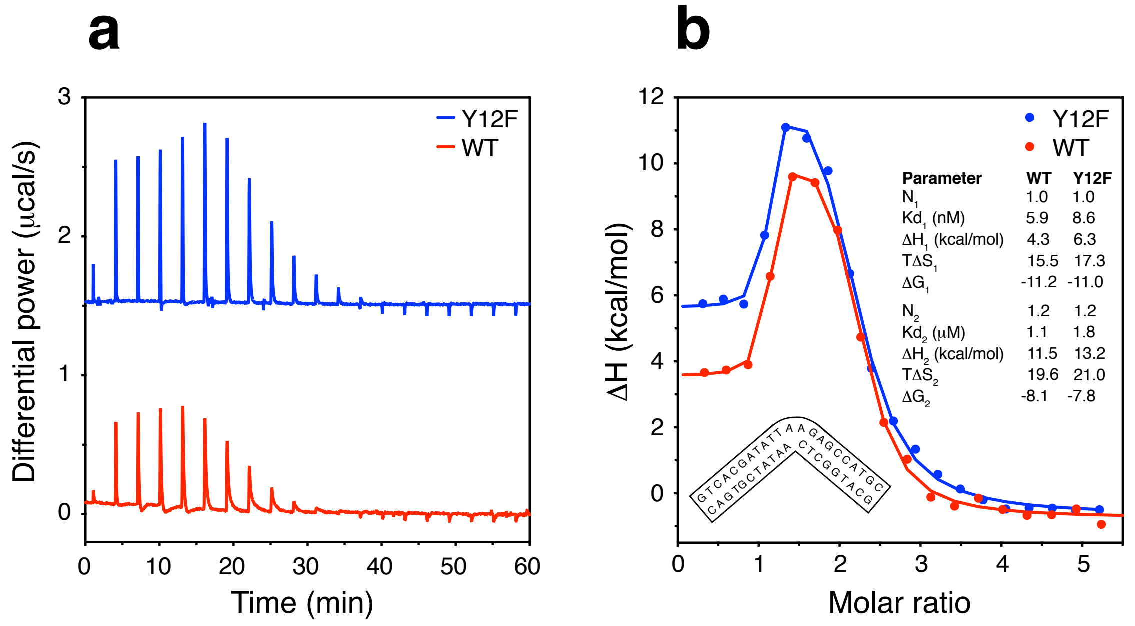

Figure S7: ITC binding isotherms for HMG-D Y12F and HMG-D WT titrated into DNA.

a) Original raw data, traces offset vertically for clarity.

b) Integrated and normalised heats (the non-sigmoidal shape indicating >1 binding site) and fits to a two-site model.

In both a) and b) data for full-length HMG-D Y12F are shown in blue, data for full-length HMG-D WT in red. The fitted binding stoichiometry (N), K_d, ΔH, TΔS and ΔG values for each site, as well as the sequence of the 22:20 dA_2 bulge DNA ligand that was used, are inset in b).

For both proteins, the primary (specific) binding site is ~200-fold tighter than the weaker one, which likely arises due to additional non-specific electrostatic interactions with exposed DNA phosphates distal to the primary binding site. The results show clearly that the Y12F mutation causes only small changes in the binding thermodynamics, and that the ΔG values in particular are very similar to one another between WT and Y12F. This similarity is evidently due to enthalpy/entropy compensation, with a slightly reduced enthalpic contribution for Y12F being largely compensated by a slightly increased entropic contribution. The enthalpic loss is likely due, at least in part, to loss of the intermolecular H-bond with the sidechain OH group of Tyr (c.f. main paper Figure 1b), while the entropic gain may be due, at least in part, to increased mobility of the Phe sidechain relative to that of Tyr. Both the primary and much weaker secondary binding to the DNA are entropically driven, with resultant affinities in the low nM and μM ranges respectively, similar to results for other HMG box proteins binding to DNA previously described by Dragan *et al.*[21]

Table S1: Numbers of intermolecular NOE-based distance restraints reported in different structure determinations of HMG box protein complexes with DNA.

Protein	Residue 12 (HMG-D numbering)	Binding class [a]	Intermolecular NOEs
HMG-D [b]	Tyr	NS	5 [c] 1 [d]
HMG-D Y12F [e]	Phe	NS	63 [c] 2 [f]
SRY [g]	Phe	S	75 [h]
SRY [i]	Phe	S	168
LEF1 [j]	Phe	S	332
NHP6A [k, l, m]	Tyr	NS	82 [l]
SRY - HMGB1 box B [n, o]	Phe (SRY);<br>Phe (HMGB1 box B)	S (SRY);<br>NS (HMGB1 box B)	46 [p, c] 54 [p, f]

[a] NS, non-sequence-specific DNA binding class; S, sequence-specific DNA binding class.
[b] Cerdan *et al.*, 2001.[6]
[c] Unambiguous restraints.
[d] Assigned in conjunction with preliminary structures.
[e] This work.
[f] Restraints applied ambiguously
[g] Werner *et al.*, 1995.[15]
[h] Number reported in pdb 1HRY.
[i] Murphy *et al.*, 2001.[16]
[j] Love *et al.*, 1995.[17]
[k] Allain *et al.*, 1999.[18a]
[l] Masse *et al.*, 2002.[18b]
[m] In this study ^{13}C,^{15}N labelled DNA[22] was employed to aid assignment of intermolecular NOEs.
[n] Structure of a DNA complex of a tandem construct of two HMG box domains; SRY and HMGB1 box B.
[o] Stott *et al.*, 2006.[19]
[p] Total for both boxes.

Table S2: Intermolecular NOE-based distance restraints used in the calculation of the NMR structural ensemble of HMG-D Y12F bound to 14-12 dA$_2$ bulge DNA.

Protein residue	Protein atom [a]	DNA nucleotide	DNA atom	Upper Bound [b] (Å)
Lys 6	Hε*	Cyt 19	H4'	5.0
Lys 6	Hδ*	Ade 20	H3'	3.5
Lys 6	Hε*	Ade 20	H3'	3.5
Arg 7	Hγ*	Thy 18	H1'	5.0
Arg 7	Hβ*	Thy 18	H1'	5.0
Arg 7	Hδ*	Thy 18	H1'	2.9
Arg 7	Hδ*	Thy 18	H4'	5.0
Arg 7	Hβ*	Cyt 19	H1'	3.5
Arg 7	Hγ*	Cyt 19	H1'	3.5
Arg 7	Hδ*	Cyt 19	H1'	2.9
Arg 7	Hδ*	Cyt 19	H3'	5.0
Arg 7	Hδ*	Cyt 19	H4'	3.5
Leu 9	Hδ*	Ade 8/Ade 9 [c]	H2	5.0
Leu 9	Hδ*	Cyt 19	H1'	5.0
Leu 9	Hδ*	Cyt 19	H3'	5.0
Leu 9	Hδ*	Cyt 19	H4'	5.0
Leu 9	Hδ*	Ade 20	H1'	2.9
Leu 9	Hδ*	Ade 20	H3'	3.5
Leu 9	Hδ*	Ade 20	H4'	3.5
Leu 9	Hδ*	Ade 21	H3'	5.0
Ser 10	Hα /Hβ* [c]	Ade 8/Ade 9 [c]	H2	5.0
Phe 12	Hζ	Thy 7	H1'	5.0
Phe 12	Hε*	Thy 7	H1'	5.0
Phe 12	Hε*	Ade 8	H1'	5.0
Phe 12	Hε*	Ade 8	H3'	5.0
Phe 12	Hε*	Ade 8	H4'	2.9
Met 13	Hε*	Ade 20	H1'	3.5
Met 13	Hε*	Ade 20	H2	3.5
Met 13	Hε*	Ade 20	H3'	5.0
Met 13	Hε*	Ade 21	H2	3.5
Met 13	Hε*	Ade 21	H1'	2.9
Met 13	Hε*	Ade 21	H4'	3.5
Leu 16	Hδ*	Thy 6	H1'	5.0
Leu 16	Hδ*	Thy 7	H1'	5.0
Leu 16	Hδ*	Ade 21	H2	5.0
Val 32	Hγ*	Ade 21	H1'	3.5
Val 32	Hγ*	Ade 21	H2	3.5
Val 32	Hγ*	Ade 21	H4'	5.0

Val 32	Hγ*	Ade 22	H1'	3.5
Thr 33	Hβ	Ade 5	H1'	5.0
Thr 33	Hγ2*	Ade 5	H1'	2.9
Thr 33	Hγ2*	Ade 5	H4'	3.5
Thr 33	Hβ	Thy 6	H1'	3.5
Thr 33	Hγ2*	Thy 6	H1'	2.9
Thr 33	Hγ2*	Thy 6	H4'	3.5
Thr 33	Hγ2*	Thy 6	H6	5.0
Thr 33	Hγ2*	Ade 21	H2	5.0
Ala 36	Hα	Thy 6	H1'	5.0
Ala 36	Hβ*	Thy 6	H1'	2.9
Ala 36	Hβ*	Thy 6	H4'	3.5
Ala 36	Hβ*	Thy 6	H6	5.0
Ala 36	Hα	Thy 7	H1'	5.0
Ala 36	Hβ*	Thy 7	H1'	2.9
Ala 36	Hβ*	Thy 7	H4'	3.5
Ala 36	Hβ*	Ade 21	H2	3.5
Lys 37	Hε*	Thy 6	H4'	3.5
Lys 60	Hε*	Gua 10	H1'	5.0
Lys 60	Hε*	Gua 10	H3'	5.0
Lys 60	Hε*	Gua 10	H4'	5.0
Lys 60	Hε*	Ade 11	H1'	5.0
Lys 60	Hε*	Ade 11	H3'	5.0
Lys 60	Hε*	Ade 11	H4'	3.5
Lys 60	Hε*	Gua 12	H4'	5.0
Tyr 63	Hε*	Ade 11	H4'	3.5
Tyr 63	Hε*	Gua 12	H4'	3.5

[a] The * symbols are wildcards indicating equivalent groups of protons; in the absence of stereospecific assignments, restraints were applied ambiguously to both atoms of prochiral methylene groups and both prochiral methyl groups of Leu and Val residues.

[b] The upper bounds shown are those used in the XPLOR-NIH stage of the calculations; these lack the additional distance corrections to allow for signal multiplicities used during the AMBER calculations where r^{-6} averaging is used rather than r^{-6} summation.

[c] These restraints were defined as ambiguous.

Table S3: Putative assignments for intermolecular NOE cross-peaks in the filtered NOESY spectrum of the complex of HMG-D WT bound to 14-12 dA_2 bulge DNA. [a]

	Protein assignments [f]	DNA assignments					
		Thy 6 H1'	Thy 7 H1'	Ade 8 H2 / Ade 9 H2 / Ade 21 H2	Ade 20 H1'	Ade 20 H2	Thy 18 H1' / Thy 19 H1' [g]
	**Arg 7 Hδ**						
**X-peaks [b]**	**3.22**						**6.08**
WT (orig.) [c]	U						6.06 / 6.17
Y12F [d]	3.20						6.01 / 6.04
Intensity [e]							medium
	**Leu 9 Hδa/Hδb**						
**X-peaks [b]**	**0.84 / 0.80**				**6.10**		
WT (orig.) [c]	0.82 / 0.82				U		
Y12F [d]	0.84 / 0.80				6.10		
Intensity [e]					weak		
	**Met 13 Hε**						
**X-peaks [b]**	**1.51**		**5.77**				
WT (orig.) [c]	U		5.74				
Y12F [d]	1.68		5.69				
Intensity [e]			weak				
	**Leu 16 Hδa/Hδb**						
**X-peaks [b]**	**0.66 / 0.38**					**7.47**	
WT (orig.) [c]	1.07 / 0.48 [h]					U	
Y12F [d]	0.65 / 0.36					7.48	
Intensity [e]						medium	
	**Thr 33 Hγ2**						
**X-peaks [b]**	**1.17**	**5.98**	**5.77**	**7.77**		**7.47**	
WT (orig.) [c]	1.17	5.98	5.74	U / U / 7.76		U	
Y12F [d]	1.27	5.93	5.69	7.68 / 7.26/ 7.90		7.48	
Intensity [e]		medium	medium	weak		weak	
	**Ala 36 Hβ**						
**X-peaks [b]**	**1.28**	**5.98**	**5.77**	**7.77**			
WT (orig.) [c]	U	5.98	5.74	U / U / 7.76			
Y12F [d]	1.50	5.93	5.69	7.68 / 7.26/ 7.90			
Intensity [e]		weak	medium	weak			

[a] This table shows the data on which the 11 intermolecular cross-peak assignments shown in Supp. Figure S4a are based. In each case, there is a close match between the chemical shift observed in the filtered NOESY spectrum and the chemical shift(s) reported in either the original deposition for the WT complex (Cerdan et al. 2001[6]), or that for the Y12F complex (present work), or both. Importantly, for any pair of cross peaks sharing a common assignment in one dimension, those cross peaks align exactly in the filtered NOESY spectrum. Where multiple values are shown for the DNA chemical shift for a given cross-peak, the corresponding restraint was applied ambiguously to all of the listed DNA protons during structure calculations of the model of the WT complex shown in Supp. Figure S4b.

[b] Chemical shifts in ppm as observed directly in the filtered NOESY spectrum shown in Supp. Figure S4a; values were calibrated by setting the methyl signal of Thr33 Hγ2 to 1.17 ppm (the value reported in BMRB 4734).

[c] Chemical shifts in ppm as reported in BMRB 4734, the original deposition for the WT complex; "U" indicates that no assignment was made for the corresponding signal in BMRB 4734. Note that chemical shift matches of the cross-peaks seen in Supp. Figure S4a with BMRB 4734 may not be exact as the present NOESY experiments were conducted with a different sample and at a temperature 3 °C warmer than for the experiments used for BMRB 4734.

[d] Chemical shifts in ppm as reported in BMRB 34874, the deposition for the Y12F complex associated with the present paper.

[e] Cross-peak intensity category, as used in the structure calculations of the model of the WT complex shown in Supp. Figure S4b. Upper limits for restraints corresponding to peaks categorised as “weak” were set to 5 Å, those corresponding to cross-peaks categorised as “medium” were set to 3.5 Å.

[f] In the absence of stereoassignments, symbols “a” and “b” indicate respectively the low- and high-field methyl signals for a given Leucine residue.

[g] In the case of the cross-peak from Arg7 Hδ, calculations specifying the DNA proton as Thy19 H1' showed significantly better convergence than others in which either Thy18 H1' alone or both Thy18 H1' and Thy19 H1’ambiguously together were specified as the DNA partner. For calculation of the model shown in Supp. Figure S4b, the corresponding restraint was defined between Arg7 Hδ and Thy19 H1'.

[h] These methyl assignments for Leu16 Hδa/Hδb in the WT complex (reported in BMRB 4734) are likely to be incorrect; comparison of the ^{13}C HSQC spectrum of the WT complex with the fully assigned ^{13}C HSQC spectrum of the Y12F complex strongly suggests that the ^{1}H shifts for WT Leu16 Hδa/Hδb are 0.64 ppm and 0.40/0.36 ppm (the higher field methyl peak is doubled in the WT spectrum).

Table S4: DNA protons in structure PDB 1E7J that lie within 5 Å of protein methyl groups assigned as showing intermolecular NOE cross-peaks for the complex of HMG-D WT bound to 14-12 dA$_2$ bulge DNA. [a]

**Cross-peaks from**	**Cross-peaks to DNA signals at**	**Candidate assignments based only on distances in structure PDB 1e7j (*r* <= 5Å)**			
Leu 9 Hδ	6.10 ppm	Ade 5 H4'<br>4.32 ppm<br>3.3 Å (6.6 ± 3.8 Å)	Ade 5 H5'<br>4.04 ± 0.27 ppm<br>3.1 Å (5.7 ± 2.8 Å)	Ade 5 H5"<br>4.01 ± 0.27 ppm<br>3.4 Å (6.1 ± 3.2 Å)	
Met 13 Hε	5.76 ppm	Thy 4 H1'<br>5.78 ppm<br>3.9 Å (5.1 ± 1.4 Å)	Thy 4 H2'<br>2.08 ppm<br>3.4 Å (4.8 ± 1.4 Å)	Thy 4 H2"<br>2.47 ppm<br>4.6 Å (6.1 ± 1.2 Å)	Ade 5 H1'<br>6.20 ppm<br>3.8 Å (4.7 ± 0.4 Å)
		Ade 5 H2<br>7.33 ppm<br>3.0 Å (3.7 ± 0.4 Å)	Ade 5 H5'<br>4.04 ± 0.27 ppm<br>4.9 Å (5.9 ± 1.0 Å)	Ade 5 H8<br>8.21 ppm<br>4.8 Å (5.9 ± 1.0 Å)	Ade 23 H2<br>7.33 ppm<br>3.2 Å (4.0 ± 1.1 Å)
Leu 16 Hδ	7.47 ppm	Ade 5 H2<br>7.33 ppm<br>5.5 Å (5.9 ± 0.5 Å)	Thy 6 H4'<br>4.15 ± 0.29 ppm<br>5.3 Å (7.2 ± 0.8 Å)		
Thr 33 Hγ2	7.77 ppm<br>7.47 ppm<br>5.98 ppm<br>5.77 ppm	Thy 7 H1'<br>5.74 ppm<br>3.0 Å (3.8 ± 0.8 Å)	Thy 7 H2'<br>1.75 ppm<br>3.8 Å (5.0 ± 0.9 Å)	Thy 7 H4'<br>4.15 ± 0.29 ppm<br>3.6 Å (5.2 ± 1.3 Å)	Thy 7 H6<br>7.08 ppm<br>4.6 Å (6.2 ± 0.7 Å)
		Thy 7 H7<br>1.73 ppm<br>3.0 Å (8.3 ± 1.8 Å)	Ade 20 H2<br>7.33 ppm<br>2.5 Å (3.1 ± 0.8 Å)	Ade 21 H2<br>7.76 ppm<br>2.8 Å (3.5 ± 0.7 Å)	
Ala 36 Hβ	7.77 ppm<br>5.98 ppm<br>5.77 ppm	Ade 5 H2<br>7.33 ppm<br>4.8 Å (5.4 ± 0.4 Å)	Ade 21 H2<br>7.76 ppm<br>4.9 Å (5.7 ± 0.4 Å)	Thy 22 H1'<br>5.61 ppm<br>2.5 Å (2.8 ± 0.3 Å)	Thy 22 H4'<br>4.15 ± 0.29 ppm<br>2.8 Å (3.6 ± 0.5 Å)
		Thy 22 H2'<br>1.99 ppm<br>3.0 Å (3.8 ± 0.7 Å)	Thy 22 H2"<br>2.33 ppm<br>4.8 Å (5.3 ± 0.6 Å)	Ade 23 H5'<br>4.04 ± 0.27 ppm<br>3.7 Å (4.4 ± 0.7 Å)	

[a] For each candidate DNA proton assignment, the corresponding chemical shift reported in BMRB 4734 is shown in black, or, in cases where no corresponding chemical shift is reported in BMRB 4734, the average and standard deviation for the corresponding proton type as reported in the BMRB statistics table for DNA protons is given in light blue (the "filtered" average, as defined by the BMRB, is shown so as to exclude outliers caused by, for example, paramagnetic effects; see https://bmrb.io/ref_info/csstats.php?restype=dna&set=filt). In each case, the distance within PDB 1E7J that would correspond to the candidate DNA proton assignment is shown in red; these distances were calculated using r^{6} averaging for distances to equivalent groups (i.e. the methyl groups, assuming no stereoassignments are available) using the program XPLOR-NIH,[8] and in each case the lowest single distance of any ensemble member in PDB 1E7J is reported, followed by the mean and standard deviation across the corresponding distances in all 10 ensemble members.

To be considered plausible, assignments require *both* that the chemical shift observed in the filtered NOESY spectrum should nearly match that of the candidate shift found in BMRB 4734 (the match may not be exact as the present NOESY experiments were conducted with a different sample and at a temperature 3 °C warmer than for the experiments used for BMRB 4734), *and also* that the corresponding distance in PDB 1E7J be short enough to be compatible with the observed NOE intensity.

No self-consistent set of assignments can be made on the basis of these data for the 11 intermolecular NOE cross peaks shown in the filtered NOESY spectrum of the complex of HMG-D WT bound to 14-12 dA$_2$ bulge DNA (Supp. Figure S4a). In particular, no plausible assignments based on 1E7J exist for the observed intermolecular cross peaks involving Leu9 or Leu16. Matching assignments do exist for cross peaks to Thr33 Hγ2 (this is not unexpected, since 1E7J was calculated using restraints from Thr33 Hγ2 to some of these DNA protons) and Met13 Hε, and possibly also for cross peaks to Ala36 Hβ, albeit corresponding to longer distances.

SUPPLEMENTARY REFERENCES:

[1] a) D. N. M. Jones, M. A. Searles, G. L. Shaw, M. E. A. Churchill, S. S. Ner, J. Keeler, A. A. Travers, D. Neuhaus, *Structure* **1994**, *2*, 609-627; b) M. E. Churchill, D. N. Jones, T. Glaser, H. Hefner, M. A. Searles, A. A. Travers, *EMBO J* **1995**, *14*, 1264-1275; c) D. Payet, A. Travers, *J Mol Biol* **1997**, *266*, 66-75.

[2] D. S. Wishart, Bigam, C. G., Yao, J., Abildaard, F., Dyson, H. J., Oldfield, E., Markley, J. L., Sykes, B. D., *J. Biomol. NMR* **1995**, ***6***, 135-140.

[3] T. D. Goddard, Kneller, D. G., University of California, San Francisco.

[4] U. Hommel, T. S. Harvey, P. C. Driscoll, I. D. Campbell, *J. Mol. Biol.* **1992**, *227*, 271-282.

[5] a) M. Nilges, *Proteins-Structure Function and Genetics* **1993**, *17*, 297-309; b) C. M. Fletcher, D. N. M. Jones, R. Diamond, D. Neuhaus, *J. Biomol. NMR* **1996**, *8*, 292-310.

[6] R. Cerdan, D. Payet, J.-C. Yang, A. A. Travers, D. Neuhaus, *Prot. Sci.* **2001**, *10*, 504-518.

[7] J. L. Kim, D. B. Nikolov, S. K. Burley, *Nature* **1993**, *365*, 520-527.

[8] C. D. Schwieters, J. J. Kuszewski, N. Tjandra, G. M. Clore, *J. Magn. Reson.* **2003**, *160*, 65-73.

[9] a) F. Sherman, J. W. Stewart, S. Tsunasawa, *Bioessays* **1985**, *3*, 27-31; b) A. Ben-Bassat, K. Bauer, *Nature* **1987**, *326*, 315.

[10] D. A. Case, T. E. Cheatham, 3rd, T. Darden, H. Gohlke, R. Luo, K. M. Merz, Jr., A. Onufriev, C. Simmerling, B. Wang, R. J. Woods, *J. Comput. Chem.* **2005**, *26*, 1668-1688.

[11] B. Xia, V. Tsui, D. A. Case, H. J. Dyson, P. E. Wright, *J Biomol NMR* **2002**, *22*, 317-331.

[12] R. A. Laskowski, J. A. Rullmannn, M. W. MacArthur, R. Kaptein, J. M. Thornton, *J Biomol NMR* **1996**, *8*, 477-486.

[13] a) R. Diamond, *Protein Sci.* **1992**, ***1***, 1279-1287; b) R. Diamond, *Acta Cryst. D* **1995**, ***51***, 127-135.

[14] W. L. DeLano, DeLano Scientific, Palo Alto, CA, **2002**.

[15] M. H. Werner, J. R. Huth, A. M. Gronenborn, G. M. Clore, *Cell* **1995**, *81*, 705-714.

[16] E. C. Murphy, V. B. Zhurkin, J. M. Louis, G. Cornilescu, G. M. Clore, *J Mol Biol* **2001**, *312*, 481-499.

[17] J. J. Love, X. Li, D. A. Case, K. Giese, R. Grosschedl, P. E. Wright, *Nature* **1995**, *376*, 791-795.

[18] a) F. H. Allain, Y. M. Yen, J. E. Masse, P. Schultze, T. Dieckmann, R. C. Johnson, J. Feigon, *Embo J* **1999**, *18*, 2563-2579; b) J. E. Masse, B. Wong, Y. M. Yen, F. H. Allain, R. C. Johnson, J. Feigon, *J Mol Biol* **2002**, *323*, 263-284.

[19] K. Stott, G. S. F. Tang, K. B. Lee, J. O. Thomas, *J. Mol. Biol.* **2006**, *360*, 90-104.

[20] M. P. Williamson, *Prog Nucl Magn Reson Spectrosc* **2013**, *73*, 1-16.

[21] A. I. Dragan, C. M. Read, E. N. Makeyeva, E. I. Milgotina, M. E. Churchill, C. Crane-Robinson, P. L. Privalov, *J Mol Biol* **2004**, *343*, 371-393.

[22] J. E. Masse, F. H.-T. Allain, Y.-M. Yen, R. C. Johnson, J. Feigon, *Journal of the American Chemical Society* **1999**, *121*, 3547-3548.
